# Cloud Computing Based Immunopeptidomics Utilizing Community Curated Variant Libraries Simplifies and Improves Neo-Antigen Discovery in Metastatic Melanoma

**DOI:** 10.3390/cancers13153754

**Published:** 2021-07-26

**Authors:** Amol Prakash, Keira E. Mahoney, Benjamin C. Orsburn

**Affiliations:** 1 Optys Tech Corporation, Shrewsbury, MA 01545, USA; amol.prakash@optystech.com; 2 Department of Chemistry, University of Virginia, Charlottesville, VA 22904-4319, USA; kem5tk@virginia.edu; 3 Department of Pharmacology and Molecular Sciences, Johns Hopkins University, Baltimore, MD 21205, USA

**Keywords:** neoantigen, cloud computing, immunopeptidomics, non-canonical

## Abstract

**Simple Summary:**

Peptides expressed on the cell surface can be used to distinguish between diseased and healthy cells and for precision drug targeting. Ideal targets in cancer diagnostics and therapeutic development are the result of altered peptide sequences that make it to the surface as expressed neoantigens. Identifying these peptides requires both genomics and proteomics sequencing technologies, which makes the process both expensive and challenging. We present an alternative solution where cloud computing can be used to improve and simplify current approaches.

**Abstract:**

Unique peptide neo-antigens presented on the cell surface are attractive targets for researchers in nearly all areas of personalized medicine. Cells presenting peptides with mutated or other non-canonical sequences can be utilized for both targeted therapies and diagnostics. Today’s state-of-the-art pipelines utilize complementary proteogenomic approaches where RNA or ribosomal sequencing data helps to create libraries from which tandem mass spectrometry data can be compared. In this study, we present an alternative approach whereby cloud computing is utilized to power neo-antigen searches against community curated databases containing more than 7 million human sequence variants. Using these expansive databases of high-quality sequences as a reference, we reanalyze the original data from two previously reported studies to identify neo-antigen targets in metastatic melanoma. Using our approach, we identify 79 percent of the non-canonical peptides reported by previous genomic analyses of these files. Furthermore, we report 18-fold more non-canonical peptides than previously reported. The novel neo-antigens we report herein can be corroborated by secondary analyses such as high predicted binding affinity, when analyzed by well-established tools such as NetMHC. Finally, we report 738 non-canonical peptides shared by at least five patient samples, and 3258 shared across the two studies. This illustrates the depth of data that is present, but typically missed by lower statistical power proteogenomic approaches. This large list of shared peptides across the two studies, their annotation, non-canonical origin, as well as MS/MS spectra from the two studies are made available on a web portal for community analysis.

## 1. Introduction

One of the most promising cancer immunotherapy options targets molecular entities that are expressed specifically by tumor cells that are lacking in normal cells [1,2,3,4]. The most common form of such entities are short peptides presented on the cell surface bound to human leukocyte antigen (HLA) molecules. Mutated neo-antigens, when expressed and presented on the cell surface, are attractive targets for immune checkpoint blockade therapies as well as clinical diagnostics [2]. It is well established that the loss of HLA heterozygosity (LOH) is a common occurrence in metastasis. Metastatic cells demonstrating LOH can demonstrate resistance to immunotherapies that were previously effective, which may accelerate reoccurrence [5,6]. Personalized therapies based on the discovery of new neo-antigens may be the only effective response [7,8,9].

Currently, discovery of such neo-antigens relies mainly on prediction-based algorithms using genomic information as input. This approach was first described more than two decades ago by Hunt and colleagues [10,11,12]. Today, mass spectrometry has shown promise as an unbiased platform to comprehensively discover naturally present HLA binding peptides, including those with post-translational modifications (PTMs) and mutations [13,14]. Many modern prediction algorithms for neo-antigen presentation now utilize HLA peptides identified by mass spectrometry in their models [15]. Independent of modeling, mass spectrometry studies on cancer cell lines and in melanoma tissues have identified neo-antigens utilizing databases made from nonsynonymous genetic alterations in the investigated tissues [13].

Data analysis is often the chronological bottleneck in shotgun proteomics. Unfortunately, in the case of immunopeptidome interrogation, this issue is exacerbated due to the exponential increase in the search space of the peptide sequences. For neo-antigen discovery, the database search algorithms are set to search with protein cleavage parameters that generate a peptide database by cutting the proteome at every amino acid [4,14]. A downside of this approach is that with even a limited set of PTMs, these may take many hours per sample, even on powerful servers, due to the sheer number of theoretical peptide sequences that must be considered. Furthermore, the large search space of peptide sequences generated have overall low biological relevance while also challenging traditional tools for false discovery rate (FDR) estimation that were not designed for use in this manner. For example, Wu and colleagues used a three step iterative search strategy on just the canonical human database to keep the FDR and search times reasonable [14]. To calculate FDR, most algorithms use decoy database strategies that were initially developed for tryptic peptides [16,17] and have been applied with little modification for the analysis of endogenous peptides that have markedly different physical and chemical properties. In this study, we present a decoy database creation strategy that is explicitly designed for endogenous peptides that provides a more accurate score for decoy match than standard proteomic tools.

An additional data analysis challenge is sequencing mutated peptides that are typically considered the best targets for cancer immunotherapy [2,3,18]. It has been suggested that because mutations are mostly unique to an individual, the MS data should be searched against a customized reference database built from a patient’s mutated protein sequences through exome sequencing, RNA sequencing (RNASeq), and ribosomal RNA sequencing (RiboSeq). While this approach is logical, to date only a small number of non-canonical peptides have been reported [15]. One recently suggested strategy was to allow for a higher threshold for FDR, with the authors recommending settings that allow a five times higher FDR rate than the limits typically employed in proteomics processing workflows [19]. Despite this body of work, these studies have shown only a small number of non-canonical peptides. This partial view is also confirmed by the low correlation between ribosomal RNA abundance, messenger RNA abundance, and mass spectrometry based peptide measurements [20]. At this stage, the reasons for these low numbers are not fully understood, but this does suggest a need for other advanced computational methods to re-interrogate the data. Some recent work has highlighted post-translational splicing events as a key event in neo-antigen expression [21,22] but these findings remain controversial [23]. Proteomic studies by tandem mass spectrometry (LCMS) have been, until [13] recently, studies of small numbers of samples compared to other -omics technologies [24]. In large part, this has been due to technological limitations that allow only a relatively small number of samples to be completed per unit time. Proteogenomic approaches have developed from proteomics labs that have utilized sequencing technologies in the same way. Without the statistical power inherent in a larger n, it is much easier to miss low copy number variants and more difficult to distinguish true signal from noise [25].

Furthermore, even though these searches are powered by a customized database, which is often quite small, these searches still had to be restrictive for their choice of PTMs and use a stringent RNAseq quality filter to ensure reasonable data searching times. On the other end of the spectrum, enormous libraries of human sequence variants have been assembled and curated from thousands of individual genomic studies. The catalog of somatic mutation in cancer (COSMIC) began with data for only four genes and now contains curated information from over 100,000 human tumors [26,27]. Another community resource of human genetic variants is maintained by the Genome Reference Consortium (GRC) and is the source of arguably the most comprehensive and high quality assembly of the human genome [28,29]. These vast libraries of human genomic variants are utilized by genomics researchers around the world, but the sheer size of these libraries restricts their utilization by proteomic researchers.

Today, cloud computing has become seamlessly integrated into our daily lives. Activities such as banking, email, media streaming, and ecommerce all use high performance cloud servers, with the user laptop or mobile being a thin interaction device. While the same model is also present in many mass spectrometry labs through remote engines such as Mascot, most of the current informatics pipelines require extensive data transfers between acquisition computers and remote servers [30]. Typically, however, the vast majority of proteomics data analysis in the world is performed on desktop computer architecture. Proteomics researchers have been historically limited in the number of sequences or post-translational modifications that can be searched due to the limitations of desktop architecture. We have recently described Bolt, a cloud-based search engine that uses scalable remote servers to search proteomics data against vast databases containing millions of protein sequences even when considering 41 common human PTMs [31,32]. Bolt is similar to consumer cloud computing tools, with a light graphic user interface that handles both upload from and presentation of the processed results to a user PC with internet connection, converting LCMS data to results in a few minutes for most files. The user provides vendor instrument raw file(s) to a Bolt app on a PC, which then creates and uploads a small binary file containing de-noised and compressed LCMS information. The Bolt engine then runs on a high-performance cloud server using in-memory indexing on a highly parallelized search algorithm to return processed results for interrogation. To the best of our knowledge, Bolt is the first cloud-based software for proteomics where raw data does not need to be present locally on the high-performance server to execute a search.

In this study, we describe the extension of Bolt for no enzyme searches and its application to immunopeptidomics data sets. To address the challenges of false discovery rate estimation, we describe an alternative approach utilizing a peptide shuffling decoy database that does not suffer from score over-estimation problems that are demonstrated in the standard reverse protein strategies used in proteomics. We demonstrate the efficacy of this approach by reanalyzing two publicly available melanoma HLA datasets and comparing to commonly used proteomics algorithms, MaxQuant, Sequest, Comet, and MS-GF+ [33,34,35,36]. To truly explore the depth of the sequence variants in the file, we have compiled a comprehensive human sequence library utilizing high quality sequences from UniProt, COSMIC, dbSNP, and GRC into a library of 7 million sequences previously observed in human samples (Table 1). While no other software tool, in our hands, could complete a search of these files utilizing databases of this size, we further extend this search with cloud resources to include likely post-translational modifications. The two original studies [13,20] reported no shared mutations across patients and 27 non-canonical peptides shared between at least two patient samples. In contrast, we report 4593 non-canonical peptides shared between at least two patients, and 738 non-canonical peptides shared across at least five patient samples. Even across the two studies, which were performed three years apart, we report 3258 shared non-canonical peptides with strong retention time correlation. Many of these non-canonical peptides are predicted to have good binding affinity for NetMHC and are independently verified by MaxQuant, Sequest, and Comet when added to the canonical database.

Using these tools, we present the largest collection of high-quality HLA peptide sequences to date and a proof of concept for the utilization of cloud computing resources and community curated sequence libraries to enable immunopeptidomics.

## 2. Materials and Methods

### 2.1. Data

The data sets used in this study were downloaded from PRIDE [37] ID PXD004894 [13] and PXD013649 [20]. The first data set consisted of MS analysis tissue samples from 25 patients, who were selected based on the diagnosis of metastatic malignant melanoma. LCMS data was acquired on a Q Exactive mass spectrometer (Thermo Fisher, San Jose, CA, USA) and consists of 78 raw files from HLA-I enrichment and 62 raw files from HLA-II enrichment, in total 140 raw files. As reported in the original study, and according to our findings, patient Mel15 had the most extensive immunopeptidome and had 24 raw files. One of these raw files: 20141208QEp7MiBaSAHLA-I-pMM153A was chosen at random for use as a representative RAW file for HLA-I (referred to as HLA-I-3A), and similarly, 20141220QEp7MiBaSAHLA-II-pMM153 was chosen as a representative RAW file for HLA-II (referred to as HLA-II-3) for all comparisons in this study. The other study, PXD013649, consisted of nine samples: three melanoma patient tissue-derived cell lines, four primary melanoma cell lines, and normal and lung tumor material from two lung cancer samples. One of the melanoma tissue cell lines: OD5P, which consisted of 16 raw files, was also studied using RNAseq and RiboSeq, and thus is used as a comparative analysis in this study.

### 2.2. Novel Decoy Strategy for No-Enzyme Search

Typically, FDR for database search in shotgun proteomics is calculated using reverse database search. Unfortunately, this strategy does not work for no-enzyme search, even though many search algorithm still use this strategy, as the y-water ions of the target peptide are now same as b- ions of the reverse peptide. Any abundant peptide that gives a rich fragmentation will also have many y ions that show water loss (Details and examples are provided in the Supplementary Material (Text S1, Table S2, Figure S8)). These reverse sequence PSMs are not truly random, but rather an artifact of how the decoy database is being created. While previous works have reported modifications to the decoy strategy, the motivations for these works has primarily been to create a decoy space that has a different amino acid or mass distribution in the enzymatic digestion-based shotgun proteomics. Thus, we devised a novel FDR strategy where we create a partial-mirror-reverse sequence for each of the peptide sequences from the target database to be used as a decoy database. Details and benefits of this strategy are described in detail in the Supplementary Materials (Text S1, Figure S9).

### 2.3. Software Comparison for Canonical Database on a High-Performance Server

We chose these software tools for comparison for the following reasons: MaxQuant [34], was used in the original publication, Sequest [36], is the most commonly used proteomics search engine, and Comet [33], and MS-GF+ [35], are two of the most recently developed and popular search engines. Each of the five software tools were installed on an Azure cloud VM, (Microsoft, Redmond, WA, USA) instance having 128 GB RAM and dual 12-core Xeon CPUs. Software version details are provided in the Supplementary Material (Text S1). For all five software tools, we used the same FASTA file used in the original dataset consisting of 85,919 Human proteins from UniProt and 245 common contaminants [38]. The comparison was performed by the analysis of two raw files: HLA-I-3A and HLA-II-3, both of which were available on the local hard drive of the server. N-terminal acetylation (42.01 Da), methionine oxidation (15.99 Da), and phosphorylation (79.97 Da on serine, threonine, and tyrosine) were set as variable modifications with a maximum of 1 PTM allowed per peptide. Peptide lengths were constrained to be between 8 and 25 residues. The enzyme specificity was set as unspecific. Mass tolerance was set as 10 ppm for MS1 and 20 ppm for MS/MS. A false discovery rate of 1% was required for peptides for all five software tools. For this comparison, amino acids leucine and isoleucine were considered the same. For comparison on a typical user computer, we used an i5 laptop with 8 GB RAM, where all software were locally installed on the laptop. For Bolt, we had the Bolt client inside Pinnacle software (Optys Tech Corporation, Shrewsbury, MA, USA) on the user laptop, but it was configured to use the Bolt server described above.

### 2.4. Software Comparison for an Ultra-Large Database

The database was expanded to use all sequence databases mentioned in Table 1, in total leading to just more than seven million protein sequences. The Bolt server was then configured using this large FASTA file, and additional PTMs (besides oxidation, phosphorylation and N-term acetylation): Cysteinyl (C), Deamidation (N, Q), Pyroglutamate (Q), and an additional 333 mass modifications from UniMod, (http://www.unimod.org) (downloaded as XML). The 333 mass modifications correspond to a total of 637 mass-residue combinations. This is run on a server having 208 vCPU and 2.8 TB RAM. As the input database grows in overall size, leading to a larger search space, the corresponding decoy space also increases. Therefore, it is expected that the score requirements for the PSMs will be higher at the same FDR. This has been explained in detail in a recent publication [39]. Bolt instead implements a class-based FDR calculation strategy. It categorizes peptide identifications into two categories: canonical and non-canonical. Both target and decoy peptides that belong to the canonical proteins get a separate FDR training vs. the ones that are non-canonical. This idea has been previously suggested, but to the best of our knowledge there is no commercial or open search engine today that implements it within its scoring routine. The end result is that Bolt is not at a disadvantage due to this increase in the search space.

**Table 1:** A summary of the protein sequences utilized in this study.

Protein Database	Number of Protein Sequences	Version/Date/Source
Human SwissProt; Canonical + isoforms	42,414	UniProt, September, 2019
Human UniProt Trembl	53,211	UniProt, September, 2019
Common contaminants	269	cRAP database [38])
Known somatic variants (missense + nonsense)	2,537,773	February, 2020 (Lazar Lab) [40]
Known population variants (dbSNP)	1,042,598	dbSNP, July, 2020
Annotated untranslated regions (UTRs)	1,976,327	GRCh38 Assembly, December, 2013 [41]
Frameshift translation	536,585	GRCh38
Annotated long non-coding (lncRNA)	882,732	GRCh38

## 3. Results

### 3.1. Comparison of Bolt to Desktop Based Search Engines with Small Canonical Database

Bolt and four other widely used proteomics search engines (MaxQuant, Sequest, Comet, and MS-GF+) were used to reanalyze the HLA-I-3A and HLA-II-3 data sets derived from human melanoma utilizing the same canonical human database, parameters, and computational architecture. Bolt identified more peptides at an estimated 1% FDR than all other engines with an average increase of 52% more peptides, using approximately 4% of the total computational search time (Text S1, Figure S1). A powerful feature in MaxQuant, called match between runs, leverages retention times between samples to increase peptide identification. To further add confidence to the peptide identifications found by Bolt, we compared Bolt’s result from one raw file and MaxQuant results from all the remaining raw files, which demonstrated a strong retention time correlation between the two sets (Text S1, Figure S1).

### 3.2. Use of Complete Human Variant Database

In our hands, the only other engine that could search against the total human variant library on the high performance server was Comet. This search required 28 h per data file and resulted in a 22% reduction in identified peptides compared to the canonical search. This is expected as without any special consideration, the larger search space will lead to a larger decoy space as well, thus increasing the required thresholds at 1% FDR [34]. Bolt instead has a built-in class based FDR calculation strategy, and thus is not at a disadvantage due to this increase in the search space [32]. Furthermore, due to Bolt’s architecture of being an in-memory computation algorithm, it can fully utilize all the processing nodes on the high-performance cloud computer.

### 3.3. Post Translational Modifications on Neo-Antigen Sequences

**Figure 1:**
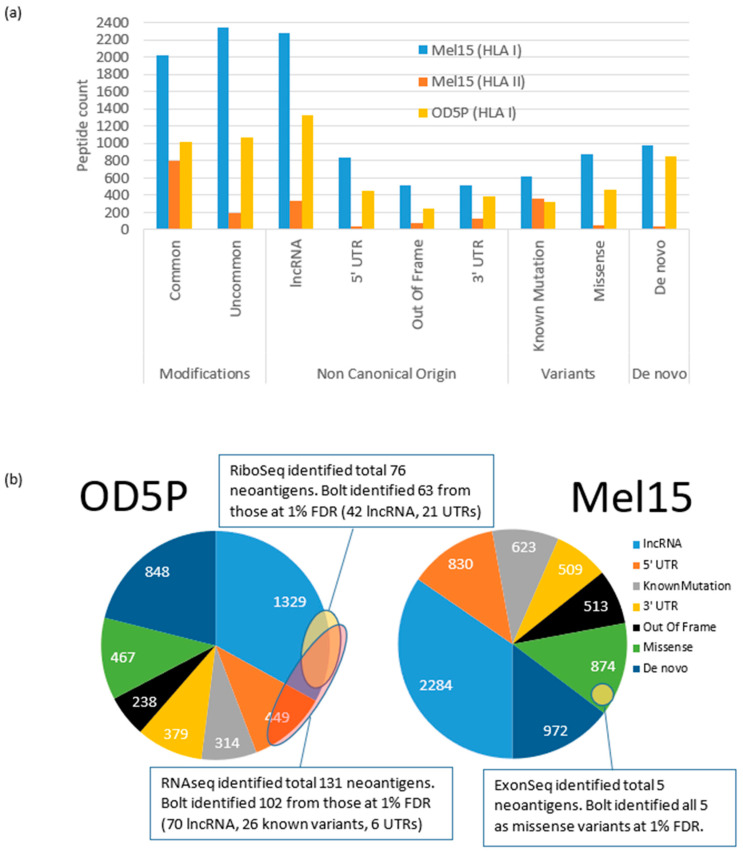
Non-canonical identifications by Bolt in the Mel15 and OD5P data sets. (**a**) Counts of various types of unique identifications in the ultra large database search. Identifications are categorized into four categories: peptides with modifications (common and uncommon), peptides from non-canonical origins, variant peptides (known and all possible missense), and de novo sequences. (**b**) Left pie chart shows the predicted neoantigens from Bolt’s results for the OD5P data set and its intersection with RNAseq and RiboSeq results. Right pie chart shows the predicted neoantigens from Bolt’s results for Mel15 data set (HLA I) and its intersection with ExonSeq results.

HLA peptides are well-established to possess post-translational modifications [35,36]. To search for the presence of PTMs in these files, we performed an all-modification search with Bolt against the human variant library (using Unimod). With a search space this large, processing each raw file HLA-I-3A and HLA-II-3 required just under 60 min each. We then compiled the results of all 24 raw files from patient Mel15 and 16 data files from OD5P. Figure 1a shows the counts of the various unique identifications observed in all 24 files from of HLA I and II sample from Mel15 as well as 16 files from OD5P and categorizes these into 4 categories: peptides with modifications (common and uncommon), peptides from non-canonical origins, variant peptides (known and all possible missense) and de novo sequences. These numbers suggest that lncRNA and 5′ UTR dominate the non-canonical peptide expression, supporting previous findings [42,43]. For the OD5P sample, 4024 peptides were identified by Bolt as non-canonical or having mutations/missense variants (as shown in the left panel of Figure 1b). In comparison, the original study identified 131 novel peptides using RNAseq and 76 novel peptides using RiboSeq generated libraries. Bolt identified 102 out of 131 (78%) of the peptide matches derived from RNASeq, and 63 out of 76 (83%) of sequences obtained by RiboSeq analysis. Combining both the RNASeq and RiboSeq libraries, Bolt identified 79% of these non-canonical peptides. Utilizing an even more stringent threshold of 0.1% FDR, Bolt identified 1437 non-canonical peptides, and this included the 79 out of 131 (60%) peptides from RNAseq and 49 out of 75 (64%) peptides from RiboSeq.

### 3.4. Identification of Variants

The original study on the Mel15 data set also utilized exome sequencing to perform stringent somatic single nucleotide variant calling, followed by creating a patient-specific custom database. This analysis identified five additional peptides with sequence variants derived from these results at a 1% peptide level FDR. As shown in the right pie chart of Figure 1b, in Bolt’s ultra large database search where all possible missense variants were considered, 1497 missense variants were identified, including the five that were reported by the original study (Table S1). In total, Bolt identified 6605 non-canonical peptides. With a more stringent threshold of 0.1% FDR, Bolt identified 2823 non-canonical peptides, including four of the five mutations identified by the exome study. Using the more stringent 0.1% FDR cutoff, we extrapolate that there are at least 18-fold more non-canonical peptides than identified by generating sequencing from the RNAseq results and 29-fold more non-canonical peptides than are found with high confidence with a single RiboSeq analysis. A comparison of the results obtained by Bolt when utilizing a canonical human database and the human variant library found that 90% of the non-canonical peptides identified were new spectral matches. Furthermore, nearly all peptides identified in the search using the canonical library were retained in both results, demonstrating that the increase in sequences searched had little FDR inflation effects (Figure S2).

### 3.5. Database Reduction to Validate Bolt Results to Desktop Search Tools

**Figure 2:**
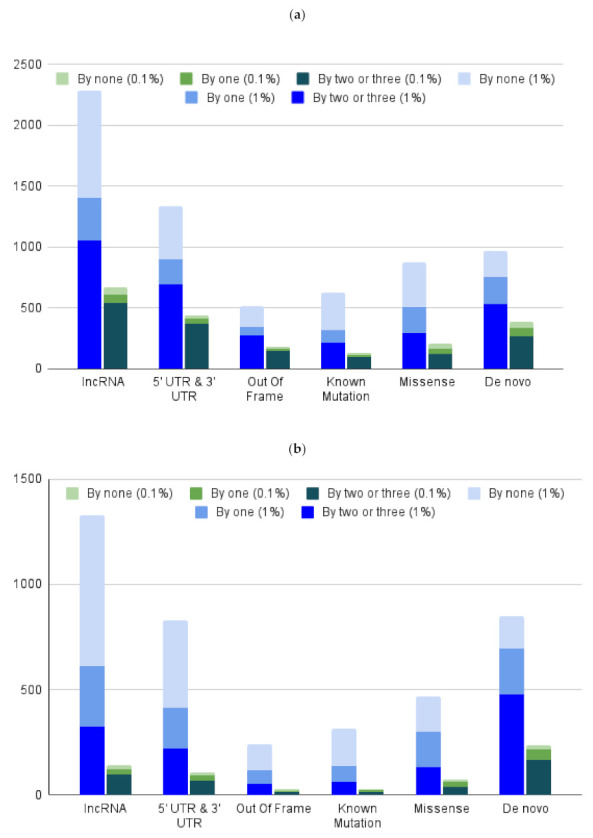
Software validation of non-canonical identifications by Bolt. (**a**) Count of Bolt’s non-canonical peptide identifications at 1% FDR for Mel15 data set that are also identified by at least two (dark blue) search engines: Sequest, MaxQuant, and Comet, one search engine (medium blue), or none (light blue). Green shows the same plot, but for 0.1% FDR identifications from Bolt. (**b**) Count f Bolt’s non-canonical peptide identifications at 1% FDR for OD5P data set that are also identified by at least two (dark blue) search engines: Sequest, MaxQuant, and Comet, one search engine (medium blue), or none (light blue). Green shows the same plot, but for 0.1% FDR identifications from Bolt.

In order to compare the results obtained by Bolt against established algorithms, we performed a reduction of the sequence variant input to enable these algorithms to function. Results obtained from Bolt using the human variant database were appended to the canonical human library. In our hands, MS-GF+ was not capable of completing this search with these settings and was not used for this analysis. Figure 2a shows the distribution of Bolt’s non-canonical peptide identifications at 1% FDR for the Mel15 data set, which are also identified by at least two (dark blue) search engines: Sequest, MaxQuant, and Comet, one search engine (medium blue), or none (light blue). In total, out of 6605 non-canonical identifications by Bolt, 64% were identified by at least one other search engine. Using a more stringent threshold of 0.1% FDR, Bolt identified 2823 peptides, of which 89% were also identified by at least one other search engine. Similarly, Figure 2b shows the same plot for the OD5P data set. In total, out of 4025 non-canonical identifications by Bolt, 57% were identified by at least one other search engine. If we use the more stringent threshold of 0.1% FDR, Bolt identified 1441 peptides, 87% of which were identified by at least one other search engine. This test lends further support to the utility of using community curated sequence libraries for immunopeptidomics. In the original study, MaxQuant reported only five mutations at 5% FDR for the Mel-15 data set, however, when utilizing Bolt’s identifications, MaxQuant identified 2493 non-canonical peptides at 1% FDR.

### 3.6. Evaluation of Neo-Antigen Sequences Identified and Genomic Context

Next, we assessed if there was a link between the expression of canonical peptides and the observed non-canonical peptides from the same coding regions (UTRs, out of frame translation, and missense). At the chromosomal level, while the majority of proteins showed only one non-canonical peptide, for the Mel15 data set, 14 proteins had four or more non-canonical peptides, and for the OD5P data set, there was one such protein (Figure S3). This analysis suggests that the expression of non-canonical peptides is not directly linked to the abundance of protein coding regions. Then, we analyzed the start and end codon characteristics of these non-canonical peptides and observed that almost 15% of non-canonical peptides end at a stop codon (Figure S4). In contrast, only 2% of peptides are derived from regions near the expected start site. Furthermore, the majority of these non-canonical peptides did not display any of the known translation initiation sites, supporting the conclusions in a recently preprinted study [44].

### 3.7. Anchor and Binding Affinity Analysis of Identified Neo-Antigens

Well-established tools exist for the analysis of neo-antigen targets based on motif and binding affinity modeling. To evaluate the results identified by Bolt in this study, we compiled all peptides with a length of nine amino acids from the two sets for these analyses. Utilizing Gibbs clustering [45], we find that these newly identified antigens present many typical anchor motifs and are therefore likely binders (Figure S5). We further assess these peptide sequences for binding affinity using NetMHC, using the recommended threshold rank of <0.5% in NetMHC 4.0 [46,47] for strong binding. Using this, we find that 1384 non-canonical peptides for the Mel15 data set and 1441 non-canonical peptides for the OD5P data set are predicted to be high binders against at least one of the HLA supertypes (Figure S5).

**Figure 3:**
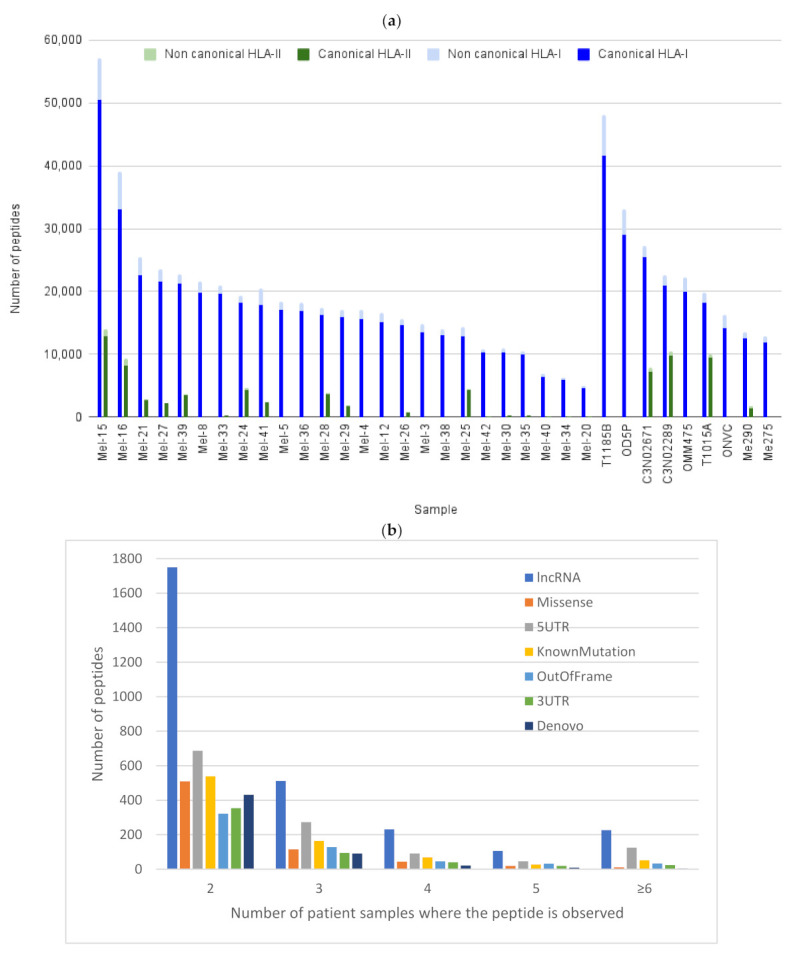
Comparing canonical and non-canonical peptides across the two data sets. (**a**) Distribution of canonical and non-canonical HLA-I and HLA-II peptides observed for each patient sample across the two data sets. (**b**) Number of non-canonical peptides observed in more than two patients plotted for the various types of non-canonical peptides.

Next, we compiled a list of all canonical and non-canonical peptides identified across the two complete data sets (total 140 + 85 = 225 data files). Bolt processed this entire data search against the ultra large database with 333 PTMs in just under 10 days and identified 407,651 canonical peptides and 54,209 non-canonical peptides. This is more than three times the number of peptide identifications reported in the original two studies. For both data sets, the non-canonical peptides from each class exhibit the expected length distribution (~9 aa for HLA-I and 14 to 16 aa for HLA-II, Figure S6). Figure 3a plots the distribution of the canonical peptide count and non-canonical peptide count observed for each of the patients for both HLA-I and HLA-II data sets (wherever available). Even though Bolt identified almost three times the number of peptides compared to the original studies, this plot models the distribution from the original studies, which also showed the number of epitopes identified per patient.

### 3.8. Conserved Neo-Antigens between Different Studies

The two original studies reported no shared missense variants across patients and 27 non-canonical peptides shared between at least two patient samples. In contrast, we identified 4593 non-canonical peptides shared between at least two patients, and 738 peptide IDs shared across at least five patient samples. Figure 3b plots this distribution of non-canonical peptide IDs observed for more than one patient sample for the various types of non-canonical peptides. No distinguishable pattern in this behavior can be discerned in this study. To expand on this, we then separated the identifications across the two data sets (one acquired in 2016 and the other in 2020) and compared the non-canonical identifications across the two. Even though these two data sets were acquired three years apart, we observed 3258 non-canonical peptides with strong retention time correlation that were identified in common across the two sets (Supplementary Figure S7). Out of these, 2826 peptides were present and identified in both studies with the same charge state, and these are available on the web portal. These results suggest that the use of small canonical libraries is a detriment to immunopeptidomics studies. Next, we listed the non-canonical and canonical peptides that were observed in both HLA-I and HLA-II. We observed that of all the HLA-I canonical peptides, approximately 6% of those are also observed in HLA-II. In contrast, only 0.6% of all the HLA-I non-canonical peptides are shared with HLA-II. Sharing of HLA-I and HLA-II peptides could be due to a cross-presentation pathway or co-purification [48]. The list of shared peptides between the two studies is available at the Bolt HLA portal [49].

## 4. Discussion

In this study we present an alternative approach to sequencing neo-antigens by leveraging pre-existing curated human sequence variant libraries. Current proteogenomic-based approaches utilize time consuming and expensive sequencing technologies to generate sequence variant libraries against which LCMS data is compared. Although commonplace today, the analysis of next generation sequencing data is a complete field of science with valuable patient cohorts often mined several times by multiple groups revealing new information with each pass [50,51]. In our approach, we utilized community curated libraries containing millions of high-quality human sequence variations. Our composite library contains approximately four million alternative sequence variations that can be tracked back to the libraries from which they are derived.

We attempted to utilize four commonly used proteomics algorithms to search published HLA peptidomics datasets against these libraries without success due to the computational limitations of software designed for a desktop PC architecture. When moving from a small canonical database to the large human variant libraries, we found a reduction in peptide numbers in other tools, due to the constraints of FDR tools designed for proteomics rather than immunopeptidomics. In contrast, the scalable cloud-based software Bolt handled these databases with relative ease even when considering as many as 333 possible mass modifications, completing these analyses in approximately one hour per file. We realize that data processing time may not be the most pressing concern of immunopeptidomics researchers, but we believe that it will be of concern if these approaches are to be truly utilized in personalized medicine in the clinic where speed is of paramount concern [52]. In addition to the scalable computational power of Bolt, we present an alternative method for development of a decoy peptide library where the decoy is generated at the peptide level rather than the protein level, using a shuffled sequence approach. The execution of these tools in tandem revealed more than 18-fold more non-canonical peptide sequences than previous analyses of these peptidomics files. Through a database reduction strategy to utilize well established proteomics tools for comparison, we found that the majority of the non-canonical peptides found by Bolt were also supported by one or more of these tools.

In addition, these peptide sequences were analyzed with well-established tools for peptide binding affinity with positive results, increasing our confidence and the value of these identifications. In our hands, Bolt was the only software that could handle such a large database with both speed and FDR constraints. All other software required significantly longer and observed a reduction in identifications due to increased search space. Without the direct use of sequencing information derived directly from the samples analyzed, Bolt identified the majority of non-canonical peptides reported from the original studies when comparing LCMS data to libraries generated from that specific sample. We find these results to be encouraging, as unique peptides expressed by an individual patient would be intrinsically less valuable as a diagnostic or therapeutic target than a neo-antigen that is found in multiple patients with a similar disease profile.

Value can still be obtained from the sequencing of the actual sample being analyzed. For example, one of the mutations reported by the 2016 study was M1482I on the gene AKAP6. This resulted in the observation of the peptide KLKLPM(M- > I)IMK. Without the genomic information for this particular strain it is impossible to determine if the MS/MS information is this sequence or another mutated canonical peptide KLKLPT(T- > I)IMK with identical mass and nearly identical MS/MS fragmentation dynamics. Another such instance is found when considering the peptides RIKQTARK and RLK457 GATARK. The first is a 5′ UTR translation, and the other is a known COSMIC mutation on H31_HUMAN. Furthermore, Q and GA are exactly isobaric, making it challenging to distinguish unless one specific fragment ion is clearly resolved. Having genomic evidence from the sample being analyzed with transcripts of either the 5′ UTR or the mutation helps clarify this peptide identity.

We also fully acknowledge that there may be other non-canonical peptides that we have not considered in Bolt. There are limitations to this approach as undiscovered mutations or peptide sequences containing multiple single amino acid variants will be missed by the approach described here. In addition, re-analysis will be necessary to consider newly discovered PTMs of importance. Even though the number of peptides observed was nearly three times larger than the original result, 40% of the spectra of these data sets are still unannotated. Notable exceptions not covered in this analysis are peptides having simultaneous mutations and multiple PTMs, or peptides with disulfide bridges that may be missed in the Bolt search. During the construction of this manuscript, a new study described a surprising observation of glycan motif deamidation of presented HLA peptide neo-antigens [18]. In the Bolt reanalysis of these files, we observe a similar pattern. Of 936 peptides identified with N deamidation for HLA-I-3A, 475 were within a glycan characteristic NXT/NXS motif, which is very similar to the 48% NXT/S motif reported and should be a target of future study.

## 5. Conclusions

The non-canonical peptide IDs by this method presents an exciting opportunity to simplify current workflows for the identification of neo-antigens. While individualized sequencing may still provide novel peptides unique to an individual or tumor, peptides identified from more than one patient are more attractive targets for diagnostics and drug development due to the ability to apply the resulting solutions to larger numbers of patients. With multiple lines of potential new cancer therapies in development today, including checkpoint inhibitors and promising developments toward cancer vaccines, the discovery of differential neo-antigens is in high demand [3,53]. Current processes, relying on combining proteomics and genomics sequencing, are more time consuming, expensive and technically challenging than either approach alone. Once peptides are identified as potential targets for checkpoint inhibitor therapy, the validation of these targets consumes additional time and resources. The result is that effective therapies are currently coupled with tremendous costs [54].

The value of community curated libraries of sequence variants is evident in most fields of research, but the constraints of proteomics software architecture has left these tools beyond the reach of mass spectrometry. A subtle shift of the paradigm to the use of scalable cloud computing, enabled by tools specifically designed to estimate FDR for endogenous peptides, allows us to utilize these valuable curated libraries for neo-antigen discovery. The application of cloud computing in this manner should lower both the cost and the development time of personalized diagnostics and therapies, particularly in the case of drug resistant metastatic events.

## Data Availability

The list of shared peptides between the two studies is available at the Bolt HLA portal [49]: http://optysbolthla.s3-website-us-west-2.amazonaws.com. To the best of our knowledge, this is the first such immunopeptidomics analysis portal that not only provides high level annotations, but also the matching details such as MS/MS spectra annotations. This portal also integrates with a previously published MS/MS annotation tool: IPSA [55]. Furthermore, it allows a user to filter by different classes of non-canonical peptides: lncRNA, UTR, known somatic or population variant, novel missense, de novo, and frameshift translation. The entire list of the peptide IDs is provided as a Supplementary.

## Supplementary Materials

The following are available online at https://www.mdpi.com/article/10.3390/cancers13153754/s1, Text S1. Supplementary Matherials and Methods, Figure S1. Comparison of different software on Mel15 and OD5P data sets on canonical database, Figure S2. Comparison between canonical database search and ultra large database search, Figure S3. Abundance comparison between canonical coding region and non-canonical coding regions, Figure S4. Study of chromosomal correlation for various non-canonical peptide IDs, Figure S5. The Anchor motif predicted by Gibbs clustering approach for the non-canonical peptides of the two data sets, Figure S6. Peptide length distribution for various classes of peptides, Figure S7. For non-canonical peptides that are observed in common across the two data sets (2016 and 2020), retention time in data set 1 is plotted against the retention time observed in data set 2, Figure S8. MS/MS spectra compared for forward and decoy peptide, Figure S9. Distribution of number of fragments matched for the reverse peptide to the same spectrum for which the target peptide had a good match (for raw file HLA-I-3A). Table S1. Mutations reported by the original study using proteogenomics approach, and their finding using Bolt’s approach, Table S2. Theoretical fragments of the peptide PKAEFAEV from the protein sequence Serum Albumin, and the corresponding peptide from the reverse protein sequence.
