# Supplementary material for: Cloud Computing Based Immunopeptidomics Utilizing Community Curated Variant Libraries Simplifies and Improves Neo-Antigen Discovery in Metastatic Melanoma
Source: Cancers (Basel). 2021 Jul 26;13(15):3754. doi: 10.3390/cancers13153754 (PMC8345142; doi:10.3390/cancers13153754)
Supplement: Supplementary file 1 [file cancers-13-03754-s001.zip › cancers-1251090-supplementary.pdf]

Supplementary Materials: Cloud Computing Based Immunopeptidomics Utilizing Community Curated Variant Libraries Simplifies and Improves Neo-Antigen Discovery in Metastatic Melanoma

Amol Prakash, Keira E. Mahoney and Benjamin C. Orsburn

1. Text S1

1.1. Detailed Software Comparison on Canonical Database

Figure 1a shows the UpSet plot comparing the results of each of the 5 software at an FDR of 1% (only the largest 20 sets are shown). Bolt and Sequest significantly outperformed all the remaining three software. In total Bolt identified 18,693 peptides, Sequest identified 15,582 peptides, MaxQuant identified 9266 peptides, MS-GF+ identified 10,730 peptides and Comet identified 11,290 peptides. The number of peptides by MaxQuant are very similar to the ones reported by the original study. Figure 1b shows the same plot for HLA-II-3 raw file, where Bolt again outperformed all the other search engines. For this raw file, Bolt identified 5424 peptides, Sequest identified 4438 peptide, Comet identified 3459 peptides, MaxQuant identified 4009 peptides and MS-GF+ identified 3111 peptides. Figure 1g shows the processing time needed by each of these software for each of these data files when the analysis file is locally present on the high performance server. Bolt outperformed all the other search engines by a significant margin by searching each of the analysis file in under 16 min. This was 4× better than Comet, 50× better than MaxQuant, 11× better than Sequest and 23× better than MS-GF+, thus on average being 22 times faster than all these search engines. Same performance gain was observed for both HLA-I-3A and HLA-II-3 raw files. Due to Bolt being a server/client architecture, when the raw file is present on a laptop, performance of Bolt was very similar as the only difference was the upload of the compressed MS/MS information to the high performance cloud server. The total time taken was just under 20 min. For MaxQuant and MS-GF+, the search took more than a day so was stopped. For Comet, the search finished in about 10 h, and for Sequest the search finished in about 14 h for HLA-I-3A and 10 h for HLA-II-3. Given these large processing times, all search engines (other than Bolt) absolutely need a high performance server to be locally present in the lab, whereas for Bolt, a lab that does not have a local high performance server can still sign up for a high performance server on Amazon AWS or Microsoft Azure, (Redmond, WA, USA), and can process their local raw files in a matter of min. For a small lab or a lab just getting started with mass spectrometry, this becomes a real need in the absence of high performance informatics server.

We then compared Bolt's results with MaxQuant, and tabulated all the peptides that were common and those that are unique to Bolt for the raw file HLA-I-3A. There were 8844 peptides in common between Bolt and MaxQuant. Out of these, 8639 peptides were also identified by MaxQuant in another raw files besides HLA-I-3A (the remaining 139 raw files). Figure 1c plots the retention time identified by Bolt in the raw file HLA-I-3A for these 8639 peptides against the retention time identified by MaxQuant in some other raw file. Then using the same idea, out of the 9849 unique identifications by Bolt, 5947 (60% of 9849) peptides were also identified by MaxQuant in some other raw file, and Figure 1d plots the retention time identified by Bolt in the raw file HLA-I-3A for these 5947 peptides against the retention time identified by MaxQuant in some other raw file. The strong correlation (R-square) and the similarity between the linear distributions further gives us confidence that the identifications by Bolt are also getting confirmed by MaxQuant in other raw files. Furthermore, from the 3012 peptides from Figure 1a that were identified only by Bolt, 1163 peptides were part of this list of 5947 peptides identified

by MaxQuant in some other raw file. Similarly, for HLA-II-3 raw file, Bolt had 3773 common and 1651 unique identifications when compared to MaxQuant. For the common identifications: Figure 1e plots the retention time identified by Bolt in the raw file HLA-II-3 for the 3618 peptides against the retention time identified by MaxQuant in some other raw file. Figure 1f plots the retention time identified by Bolt in the raw file HLA-II-3A for the 884 peptides (54% of 1651) against the retention time identified by MaxQuant in some other raw file. As discussed before, both plots show a very similar (and strong) correlation, thereby adding confidence to the Bolt's identifications. Also, from the 753 peptides from Figure 1b that were identified only by Bolt, 262 peptides were part of this list of 884 peptides identified by MaxQuant in some other raw file.

1.2. De Novo Comparison

Strategies outside of database searches, e.g., de novo search, have also shown promise in immunopeptidomics but controlling for FDR has been challenging. A recent publication showed a large set of de novo peptides from immunopeptidome cohorts [27], but a follow up reanalysis then showed most of these identifications to be false positives [22], attributing most of the issues to FDR. The later publication identified endogenous peptides with PTMs to match the corresponding MSMS spectrum. We report similar challenges with de novo searches, by comparing de novo searching by Peaks [additional ref 1] (which is one of the most popular de novo tools) versus canonical database searching by Bolt. Figure 1h shows the count of peptides that are identified by Peaks at a specific de novo ALC score for raw file HLA-I-3A, and how do the identified peptide sequence compare with the canonical identification from Bolt for the same MS/MS spectra. Even at very high score at 90 or higher, 15% of sequence assignments by Peaks are different than those by Bolt (for just the canonical database), and at score of 70 or more, 25% of assignments by Peaks are different. So even though Peaks is assigning many spectra that are unassigned by Bolt, but the exact sequence assignment has a high probability of being incorrect. This isn't a criticism of Peaks algorithm, but rather the challenge of de novo sequencing, due to MS/MS spectra often having contaminant ions from other peptide/non-peptide species as well as peptides often having incomplete fragmentation. Many of the differences between Peak's assignment and Bolt's assignment are different by just one or two amino acids, but immunopeptidome no-enzyme in-silico digestion leads to sequences with high degree of similarity to each other, and thus getting the exact sequence match is critical so as to decide whether it is canonical or non-canonical. As expected, the longer the peptide sequence, the harder it will be for de novo to get the exact sequence correct, and Figure 1i shows the similar result for raw file HLA-II-3. At a score of 70 or higher, 58% of sequence assignments are different from the ones from Bolt's canonical database search. Thus it is critical to have database search and support to ensure appropriate sequence matching to canonical and non-canonical peptides.

1.3. Comparison Between Results from Ultra Large Database and Results from Canonical Database

Using the canonical database, we identified 59,275 peptide identifications (56,531 unique sequences) for Mel15. Using the ultra-large database, this number increases to 77,686, which corresponds to 31% increase. Given that the ultra large database had more than 7 million proteoforms and 600+ modifications, observing a reasonably small, but significant increase further shows the high specificity in the results. Figure 2a compares the peptide IDs from the ultra large database (77,686) against the canonical database search (59,275 peptides) for the Mel15 data set. Between these two lists, there were 51,315 peptides in common, and their distribution of −ln(q value) is plotted in black in Figure 2a, showing that most of these are high confidence peptides. Solid grey shows the 13,698 peptides that are unique to the ultra large database search but are also available to the canonical database search, and dotted grey shows the 7960 peptides that are unique to the ca-

nonical database search, both of which have worse q value distribution. This adds confidence, as the expanded database search is capturing most of the high confidence results, and a small fraction (~13%) of low confidence identifications are being replaced by another set. Yellow shows the 4702 peptides from non-canonical origins (lncRNA, Out of frame translation and UTRs), orange shows the 4984 peptides with additional modifications (both common and uncommon) and green shows the 1905 peptides with mutations (both known and all possible missense). Similar to this, Figure 2b compares the peptide IDs from the ultra large database (29,629) against the canonical database search (28,448 peptides) for the OD5P. 23,900 peptides that are common between Bolt's canonical database search and ultra large database results are shown in black. Solid grey shows 5729 peptides that are unique to the ultra large database search but are also available to the canonical database search and dotted grey shows 4548 peptides that are unique to the canonical database search. Similar to the Mel15 data set, this shows that the expanded database search is capturing most of the high confidence results, and a small fraction (~16%) of low confidence identifications are being replaced by another similar size set. Yellows shows the 2395 peptides from non-canonical origins (lncRNA, Out of frame translation and UTRs), orange shows the 1947 peptides with modifications (both common and uncommon) and green shows the 781 peptides with mutations (both known and all possible missense).

When we compare the results on the canonical database and ultra large database on a spectrum-by-spectrum basis, we report that across the 24 raw files of Mel15, there were 34,146 spectra that led to identification of non-canonical, variants and additional modifications. Out of these 30,294 (~89% of 34,146) spectra were unassigned in the canonical database search, thereby further adding confidence that these identifications are indeed novel. From the remaining, 876 spectra are possibly chimeric, and 2976 (9% of 34,146) spectra show a conflict in the identification against the two databases. Figure 2c plots the increase in matched ion count and dot product for these 2976 spectra, for the search against the ultra large database vs. the canonical database. Most spectra showed an increase of 2 to 4 ions annotated, thus further adding trust that these peptide annotations are higher confidence compared to the canonical match from the canonical database. Similarly, for the OD5P dataset, there were 18,485 spectra that led to identification of non-canonical, variants and additional modifications. Out of these 16,749 (~91% of 18,485) spectra were unassigned in the canonical database search, thereby further adding confidence that these identifications are indeed novel. From the remaining, 262 spectra are possibly chimeric, and 1474 (8% of 18,485) spectra show a conflict in the identification against the two databases. Figure 2d plots the increase in matched ion count and dot product for these 1474 spectra, for the search against the ultra large database vs. the canonical database. Most spectra showed an increase of 2 to 4 ions annotated, thus further adding trust that these peptide annotations are higher confidence compared to the canonical match from the canonical database.

1.4. Genome Wide Analysis

We assessed if there was a correlation between the expression of canonical peptides and the expression of the observed non-canonical peptides from the same coding regions (UTRs, out of frame translation, and missense). We used two commonly used methods of representing expression: (1) sum of area under the peak of most abundant three peptides for every protein and (2) total assigned MS/MS to each protein (called spectral counting). The same were also calculated for non-canonical regions. We observed very poor correlation (R-square <0.5) for both Mel15 and OD5P data sets, for all three: UTRs, Out of frame translation and missense expression (Figure 3) suggesting that the expression of non-canonical peptides isn't a feature of high abundance coding regions.

Then we wanted to assess if there is a correlation at the chromosome level. Figure 4 plots the number of canonical peptides observed on a chromosome plotted against the number of (a) UTR (b) lncRNA (c) missense non-canonical peptides observed on the same

chromosome for Mel15 (blue) and OD5P (orange) data sets. For each of these plots, the r-square ranges from 0.79 to 0.91, which suggests that the chromosome that have a higher number of coding regions observed have a higher probability of also show non-canonical expression. To then study whether there are specific hotspots, we plotted the number of proteins against the number of non-canonical peptides of various types observed for that protein for both (d) Mel15 and (e) OD5P data sets. While majority of proteins showed only one non-canonical peptide, for the Mel15 data set there are 318 proteins that had two or more UTR, lncRNA or out of frame peptides, and 66 proteins that had two or more peptides with missense mutations. Similarly, for the OD5P data set, there are 92 proteins that had two or more UTR, lncRNA or out of frame peptides, and 26 proteins that had two or more peptides with missense mutations. For the Mel15 data set, 14 proteins had 4 or more UTR, lncRNA or out of frame peptides, and for the OD5P data set, there was 1 such protein. Next, we wanted to see if these peptides had any specific start or end codon characteristics. As the genomic UTR, lncRNA and out of frame translation regions are supposed to have specific start sites as well as stop codon can be observed during the translation event, Figure 4f plots the number of non-canonical peptides that end at a stop codon (light blue/green), or start at the expected start site (medium blue/green), and the remaining (dark blue/green). As there are only 3 codons out of 64 that generate the stop codon, observing stop codons for almost 15% non-canonical UTR, lncRNA and out of translation peptides shows that there is a higher probability of observing a non-canonical peptide near a stop codon and also suggests that their processing might be proteasome-independent. In contrast, only 2% peptides seem to start from the expected start site, suggesting there is no correlation between expected start and the observed non-canonical peptide. Furthermore, majority of these non-canonical peptides did not display any of the known translation initiation sites.

1.5. Binding Study

We then compiled all of the length 9 non-canonical peptides from the two data sets for their predictive binding. This gave 3197 peptides for the Mel15 data set and 2191 peptides for the OD5P data set. Using Gibbs clustering [28], we show that each of these sets have the typical anchor motifs and are therefore likely binders. Figure 5 shows the motifs for both of these data sets with the size "n" for each motif described in the figure caption. Another common approach to assess the performance of the peptide IDs is to estimate their binding affinity using NetMHC [24]. Strong binding threshold was set to rank <0.5% (standard setting in NetMHC 4.0). Using this, we found 1384 peptides for the Mel15 data set and 1441 non-canonical peptides for the OD5P data set that are predicted to be strong binders against one of the HLA supertypes.

1.6. Novel Decoy Strategy for No-enzyme Search

Typically, FDR for database search in shotgun proteomics is calculated using target and decoy database search. A decoy database is created that is of similar size as the target database, and all the PSMs in this database are used as decoy hits (e.g., for training Percolator). Two strategies have previously been suggested for creating decoy database [11]: (a) shuffling the protein sequence or (b) creating the reverse protein sequence. The first strategy does not work when there are many homologous sequence in the database (e.g., isoforms and variants) as this homology cannot be maintained during shuffling, and thus it will create a larger decoy search space than the target search space. The second strategy, whereby the protein sequence is reversed, is the most commonly used strategy for shotgun proteomics. Here the main principle is that by reversing the sequence, the N- and C-terminal ends of the tryptic cleavage get switched, and thus it creates a very different precursor/fragment information than the original sequence. For example, if we consider Serum Albumin protein sequence: …RFPKAEFAEVSKLV… the target peptide will be AEFAEVSK and the corresponding peptide from the reverse peptide will be SVEAFEAK.

Even though both will have the same precursor mass, their fragments will be very different due to the switching of the position of Lysine.

Unfortunately this strategy does not work for no-enzyme search even though many search algorithm still use this strategy. Let's consider any peptide from the Serum Albumin protein sequence (with no requirement of cleaving at K, R): PKAEFAEV, then the corresponding peptide from the reverse sequence is VEAFEAKP. Both of these peptides will have the exact same precursor mass (mol. wt. 889.455). Supplementary Table 2 lists the b- and y- fragments for the target peptide and reverse peptide, along with the water loss m/z for y ions. As one would expect, the y-water ions from the target peptide are exactly the same as b- ions of the reverse peptide. Any abundant peptide that gives a rich fragmentation will also have many y ions that show water loss. Figure 8 shows two such examples. Top panel of (a) shows peptide sequence KAVLTIDEKGTEA, from protein A1AT_HUMAN, having a strong match to scan number 28,033 in the raw file HLA-I-3A. The lower panel of (a) shows the same spectrum also having a rich fragment match to the reverse peptide AETGKEDITLVAK because many of the y-water ions from the target peptide are now the b ions of the reverse peptide. So by most algorithm this PSM will score fairly high. The panel in (b) show another example, with top panel for the KLFDSTTLEHQK (from protein EIF3I_HUMAN) and its reverse counterpart KQHELTTSDFLK, both having a good match to the scan number 28,223 from the same raw file. These PSMs also have the same behavior, where the abundant peptide with rich fragmentation shows many y-water loss ions, and thus these become b ions for the reverse peptide sequence. Further, to show that this observation isn't limited to a handful of peptides, we consider the same raw file, and tabulate all peptides from the forward database that match this raw file and the number of fragment PSMs for each. Then for each such PSM, we consider the corresponding reverse peptide and count the number of fragments that this reverse peptide has to the exact same spectrum. Figure 9 plots this distribution. For target peptides having fewer fragments (e.g., 4 or 5) matched to a spectrum, the reverse peptide has almost no matches to the peptide (large orange or grey bar). But for target peptides that have a rich fragmentation match to a spectrum (e.g., ≥12), a significant number of those also have the reverse peptide with a rich fragmentation match to the same spectrum. The blue and the green bars together make almost 20% of such peptides, which means almost 20% of the target peptides yield a large number of y-water loss ions which would then get counted as b ions for the reverse peptide. If instead we took shuffled or truly random sequences, this percentage should be close to zero instead of being 20%.

Thus if we simply consider reverse sequences as the decoy database, all these high scoring matches will cause the training algorithm (e.g., Percolator [additional ref 2]) to put even more stringent thresholds for the target database at a specified FDR. But in reality these reverse sequence PSMs aren't really random, but rather an artifact of how the decoy database is being created.

While previous works have reported modifications to the decoy strategy, the motivations for these works has primarily been to create a decoy space that has a different amino acid or mass distribution in the enzymatic digestion based shotgun proteomics. To the best of our knowledge, no previous study has shown this significant overestimation of random hits in the case of no-enzyme searches due to the similarity of ions between target and decoy peptide. For example, for MaxQuant, reverse protein sequences are modified by swapped every arginine and lysine with the preceding amino acid in the reversed sequences. Unfortunately, this will not correct for the over-estimation for no-enzyme searches as we need to consider cleavage at every amino acid (and not just arginine/lysine). So peptides not having arginine or lysine will create their exact reverse sequence with no such correction. Another study suggested the pseudo-reverse strategy, implemented in Sage-N and Comet, where the target peptide is reversed for all amino acids except for the N-terminal residue (to preserve the cleaving amino acid). An additional benefit of this strategy is that this create a decoy peptide with the same molecular weight as the target peptide. While this does not create an issue for a tryptic peptide (as the b-

and y- ions are very different between target and decoy), it still does create an issue for no-enzyme searches. If the composition of the peptide is dominated by one or two amino acids, or if the peptide is near-palindrome, the b- and y- ions would again be highly similar for the target and decoy peptide. Furthermore, having the exact same m/z would again cause artifacts like Figure 9, and thus cause an overestimation of scores of random hits.

Thus we devised a novel FDR strategy where we first create all possible peptide sequences from the target database. Then for each peptide we create the reverse peptide, and the last 4 residues of the reverse peptide are swapped with the mirror image of the 4 amino acids that are 20 residues downstream in the original protein sequence. If this region is still similar (e.g., in case of repeats) or we are at the C-terminal of the protein, then we instead select from the region 20 residues upstream from the start position of the peptide. This strategy has multiple advantages: (1) the protein-level amino acid distribution remains similar between target and decoy. (2) Molecular weight of the target and decoy peptide will be different thus there are very low chances of both target and decoy matching the same spectrum, even if the target peptide is a palindrome or near-palindrome. (3) A distance of 20 amino acids ensures that peptide regions that are dominated by one or two amino acids get replaced by an altogether different set of amino acids thus again reducing the odds of both target and decoy matching the same spectrum.

In our analysis, a small portion of Bolt's performance (for peptide count) is attributed to the novel decoy strategy mentioned above. When we use a simple reverse sequence based decoy strategy, which we explained will lead to overestimation of the scores or random hits, we see Bolt's peptide count to decrease by 5–7% than before. This is expected as Figure 9 shows that this observation of neutral loss ions is primarily limited to high abundance peptides that have a rich fragmentation, thus we did not expect to see this novel strategy to cause a dramatic change in number of peptide IDs. Nonetheless, even if the gain is small, the novel decoy strategy is still the more appropriate model for the decoy database to avoid overestimation.

1.7. Novel De Novo Strategy

To identify non-canonical peptides that may not be available in the database, such as hybrid peptides, Bolt implements a de novo peptide sequencing algorithm to sequence all the spectra that remain un-annotated after the ultra-large database search. To the best of our knowledge, there is currently no accepted or published FDR strategy for de novo sequencing algorithms. For Bolt's de novo search identifications we implemented a rigorous two-step FDR calculation. In the first step, a database search is performed, and all PSMs with ≤1% FDR are marked as identified. In the next step, all MS/MS are then provided to a novel de novo algorithm which considers all possible amino acid combinations to report the peptide sequence that best explains each spectrum. We do not consider PTMs in this step. Then using the database results, we can identify which de novo PSMs are incorrect (as the sequence is different than the high confidence database result), and those are marked as decoy. The rest are marked as target or forward search. These PSMs along with all the PSMs from the database search are again used for Percolator training. By doing so we obtain a q value for all the forward de novo PSMs, and only those that are above 1% FDR are used. While this might seem too stringent (i.e., to call every de novo sequence that is different from database as a decoy), this enables us to generate a very high confidence list of de novo IDs.

1.9. Software Version

MaxQuant was run inside MaxQuant (v 1.6.7.0, July 2019). We chose to re-run MaxQuant instead of using the results from the old data set as we wanted to use the latest version of MaxQuant. Also, the original data set had enabled the option 'match between runs' which can artificially inflate the identifications in a single raw file using evidence from other raw files even though it may have a higher FDR than 0.01. So we re-ran MaxQuant without this option. Comet was run via command line (v 2019010, Aug 2019),

and then FDR was calculated using Comet UI (v 1.0.0.83, Jan 2018). Sequest-HT was run inside Proteome Discoverer 2.4 and used Percolator for FDR calculation. MS-GF+ was run as a command line utility and it required that raw files be converted to mzXML using RawConverter, followed by MzidToTsvConverter for FDR calculations. Bolt server was run as a command line utility, and Bolt client was run inside Pinnacle software (v 1.0.99).

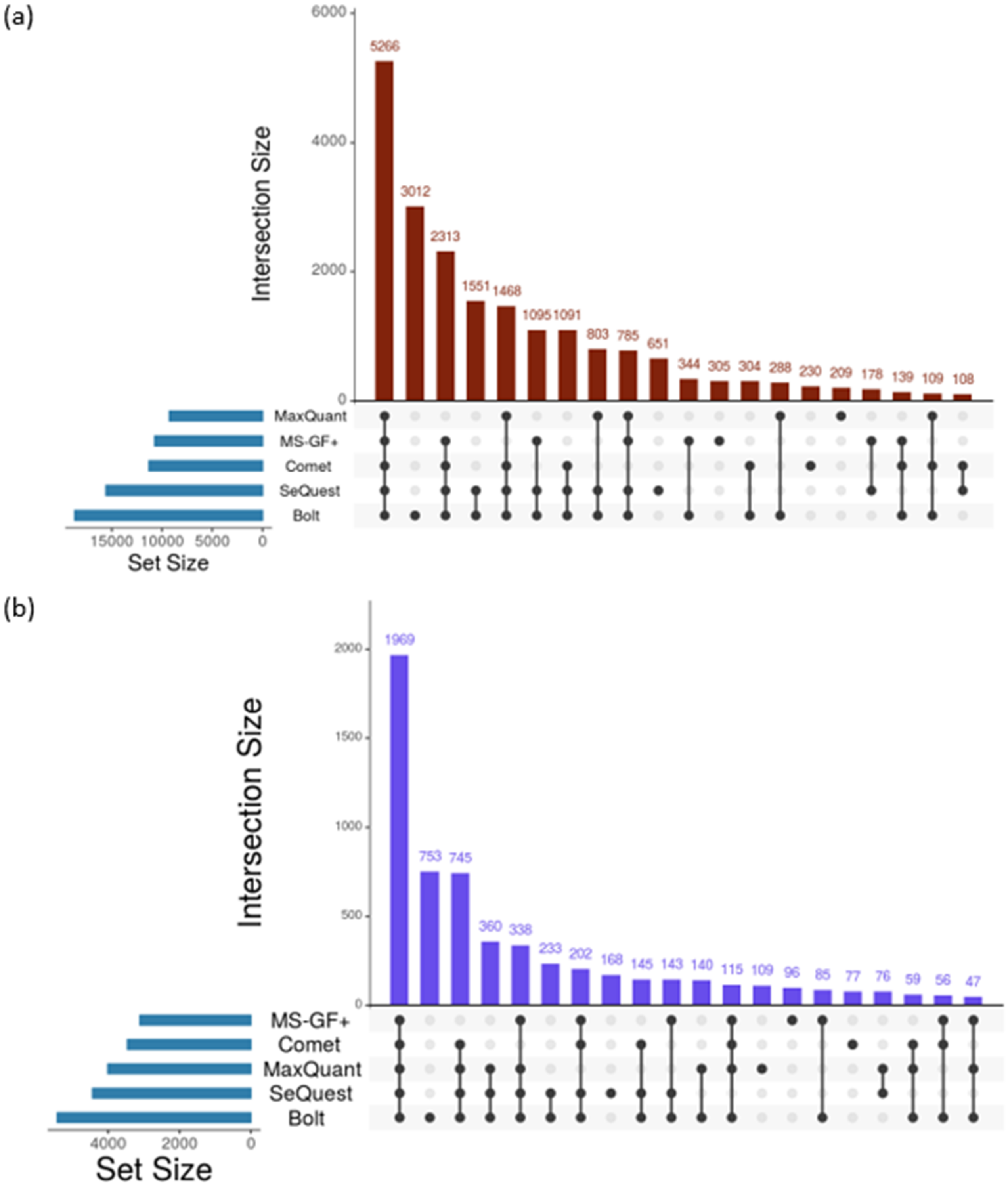
(a)
Intersection Size
6000
4000
2000
0
5266
3012
2313
1551
1468
1095
1091
803
785
651
344
305
304
288
230
209
178
139
109
108
MaxQuant
MS-GF+
Comet
SeQuest
Bolt
15000 10000 5000 0
Set Size
(b)
Intersection Size
2000
1500
1000
500
0
1969
753
745
360
338
233
202
168
145
143
140
115
109
96
85
77
76
59
56
47
MS-GF+
Comet
MaxQuant
SeQuest
Bolt
4000 2000 0
Set Size

(c)

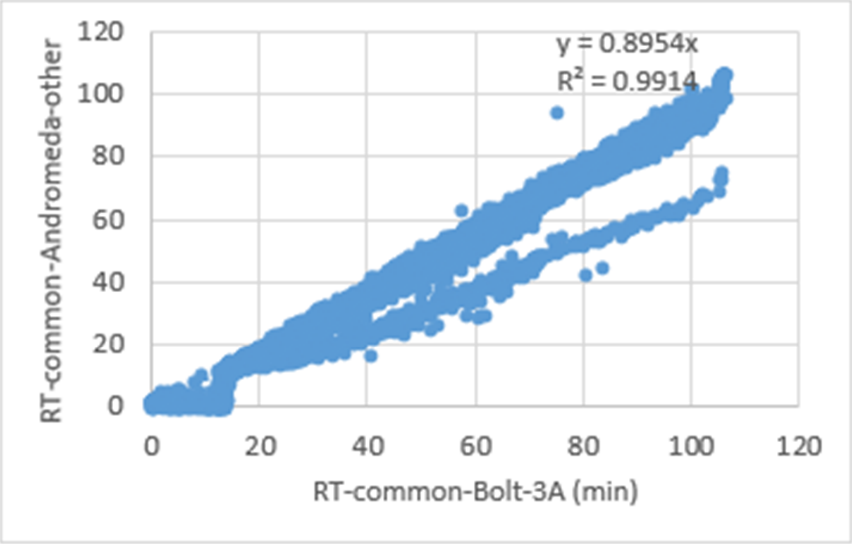
y = 0.8954x
R² = 0.9914
RT-common-Andromeda-other
RT-common-Bolt-3A (min)
0
20
40
60
80
100
120

(d)

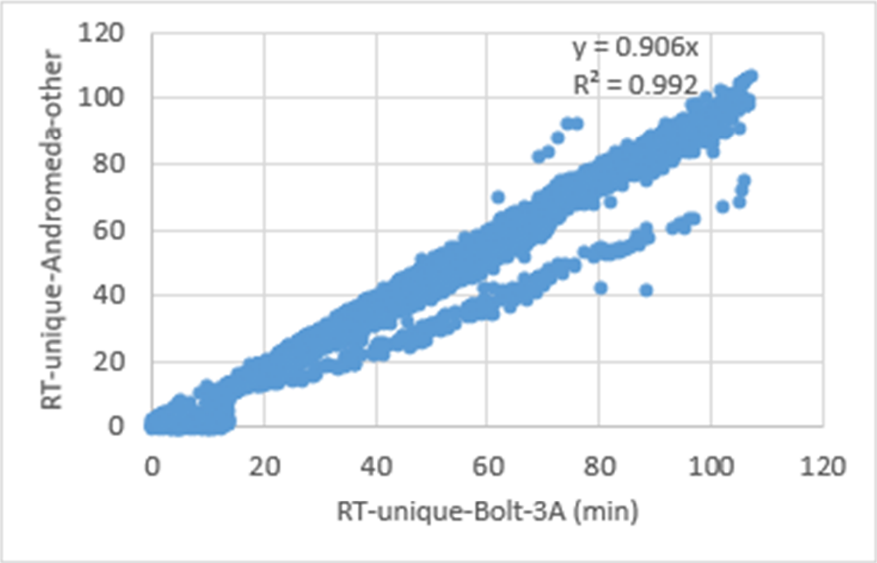
y = 0.906x
R² = 0.992
RT-unique-Andromeda-other
RT-unique-Bolt-3A (min)
0
20
40
60
80
100
120

(e)

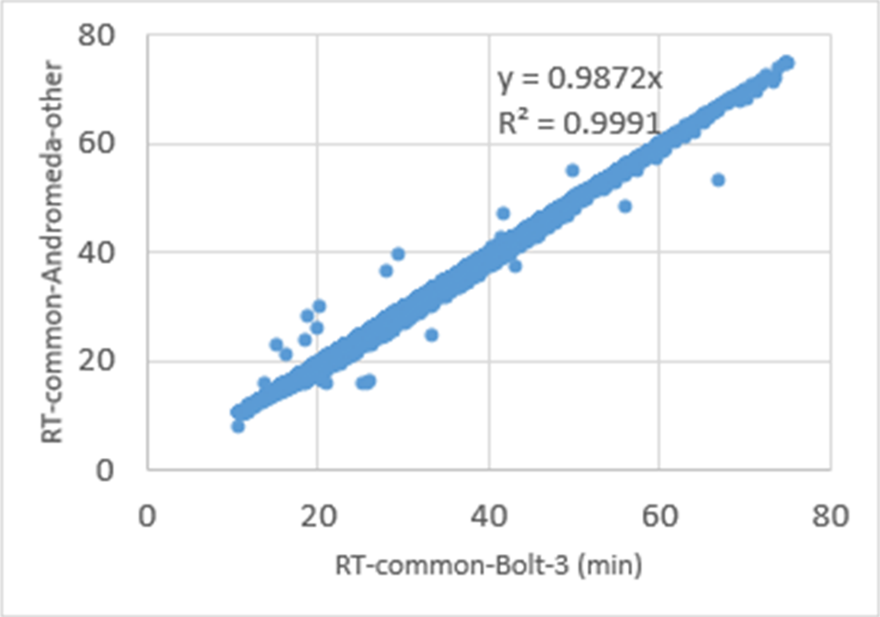
y = 0.9872x
R² = 0.9991
RT-common-Andromeda-other
RT-common-Bolt-3 (min)
0
20
40
60
80

(f)

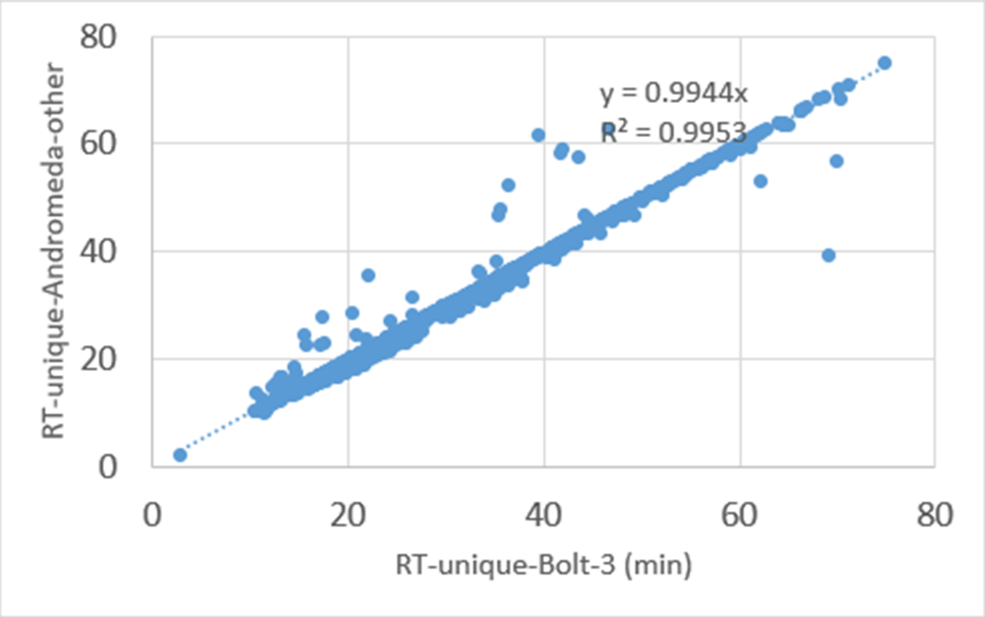
y = 0.9944x
R^2 = 0.9953
RT-unique-Andromeda-other
RT-unique-Bolt-3 (min)
0
20
40
60
80

(g)

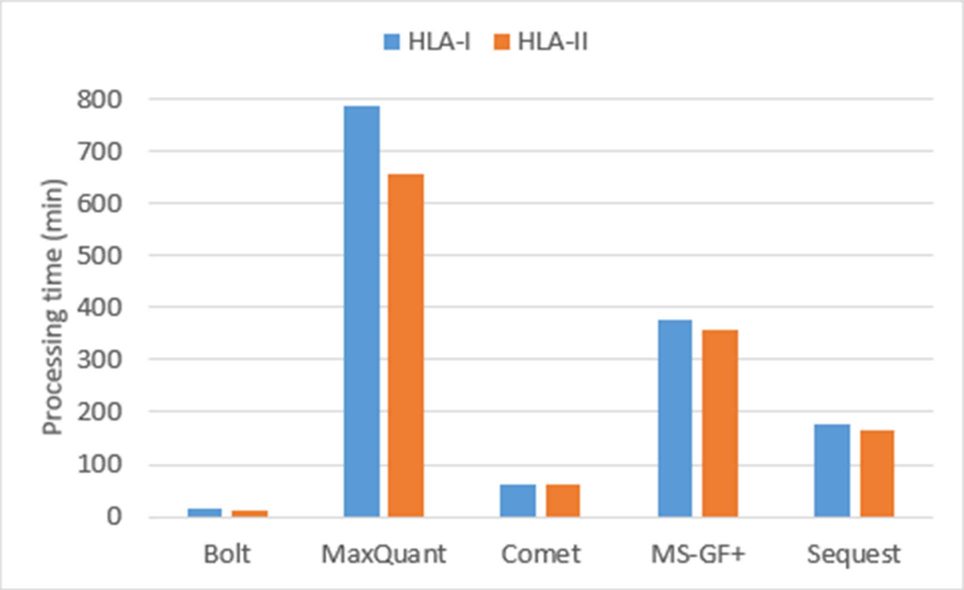
HLA-I
HLA-II
Processing time (min)
0
100
200
300
400
500
600
700
800
Bolt
MaxQuant
Comet
MS-GF+
Sequest

(h)

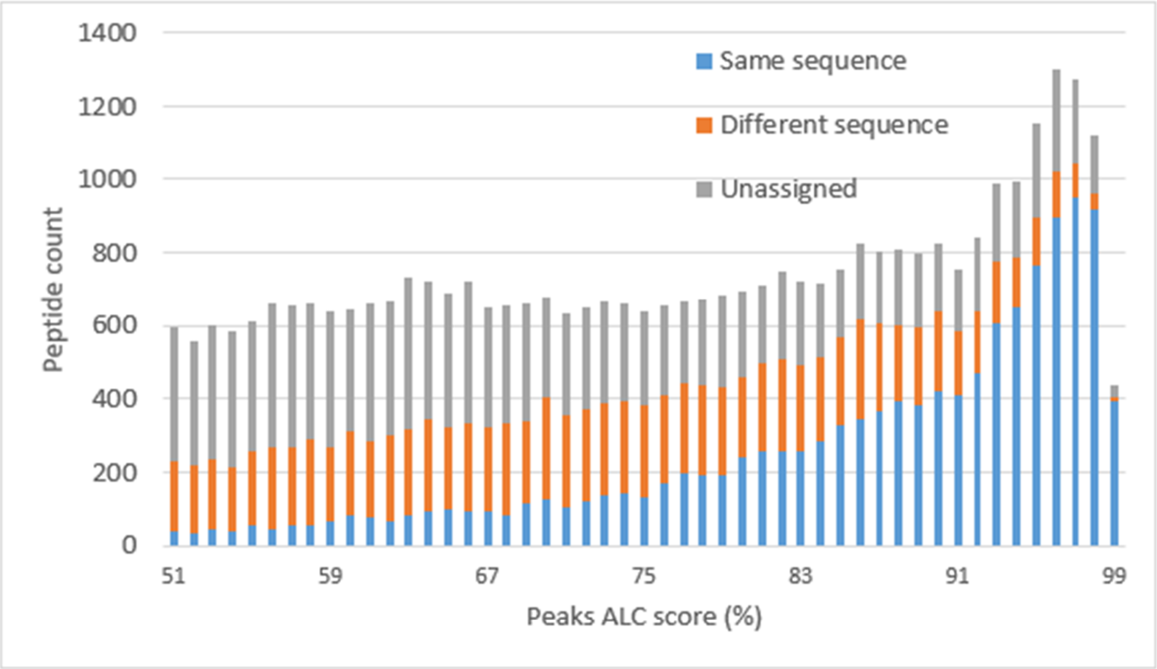

(i)

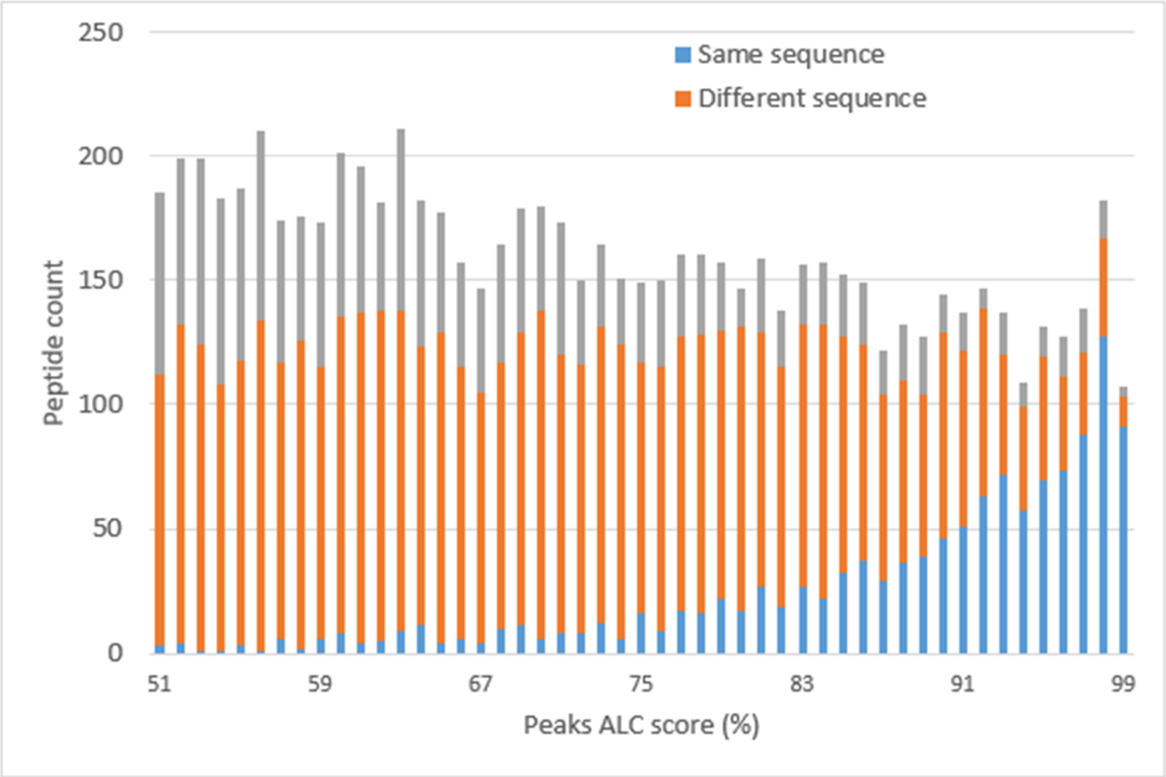

Figure S1. Comparison of different software on Mel15 and OD5P data sets on canonical database. (**a**) UpSet plot of peptides identified by each of the 5 software for the HLA-I-3A data set with FDR at 1%. (**b**) UpSet plot of peptides identified by each of the 5 software for the HLA-II-3 data set with FDR at 1%. (**c**) Comparison of retention time identified by Bolt in raw file HLA-I-3A with the retention time identified by MaxQuant in another raw file for 8639 common peptides and (**d**) 5947 unique identifications of Bolt. X axis is the retention time identified by Bolt in HLA-I-3A raw file, and the y axis is the retention time identified for the same peptide by MaxQuant in any raw file other than HLA-I-3A. (**e**) Comparison of retention time identified by Bolt in raw file HLA-II-3 with the retention time identified by MaxQuant in another raw file for

3618 common peptides and (**f**) 884 unique identifications of Bolt. X axis is the retention time identified by Bolt in HLA-II-3 raw file, and the y axis is the retention time identified for the same peptide by MaxQuant in any raw file other than HLA-II-3. (**g**) Processing time of each of the 5 software for HLA-I-3A and HLA-II-3 analysis on the high performance server. (**h**) The count of peptides that are identified by Peaks at a specific ALC score for raw file HLA-I-3A, and how do the identified peptide sequence compare with the canonical identification from Bolt for the same MS/MS spectra. (**i**) The count of peptides that are identified by Peaks at a specific ALC score for raw file HLA-II-3, and how do the identified peptide sequence compare with the canonical identification from Bolt for the same MS/MS spectra.

(a)

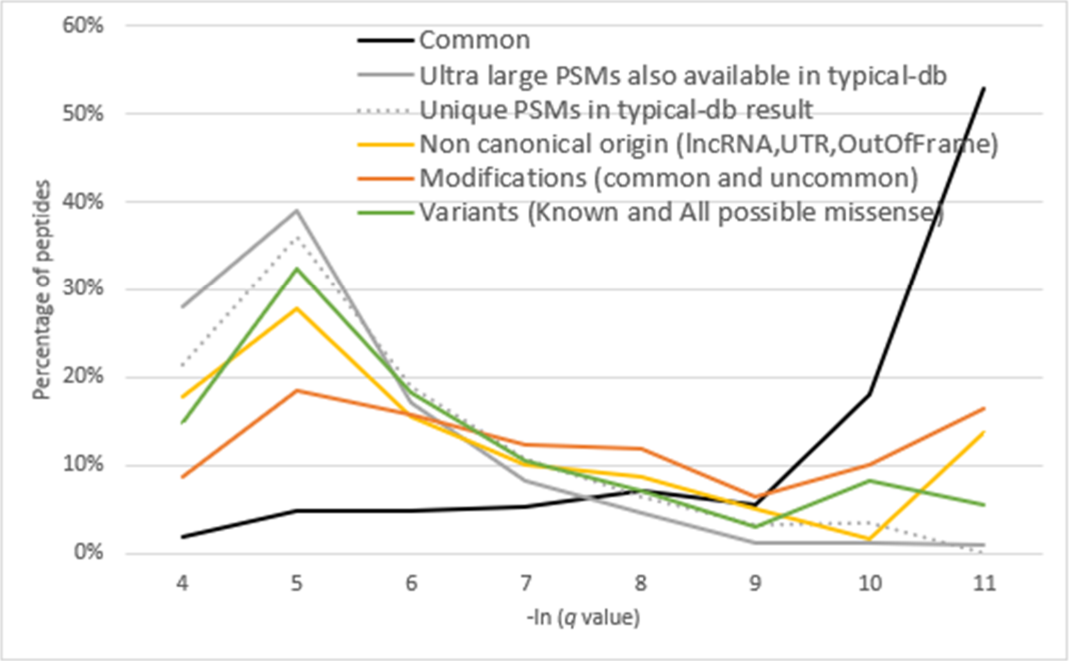

(b)

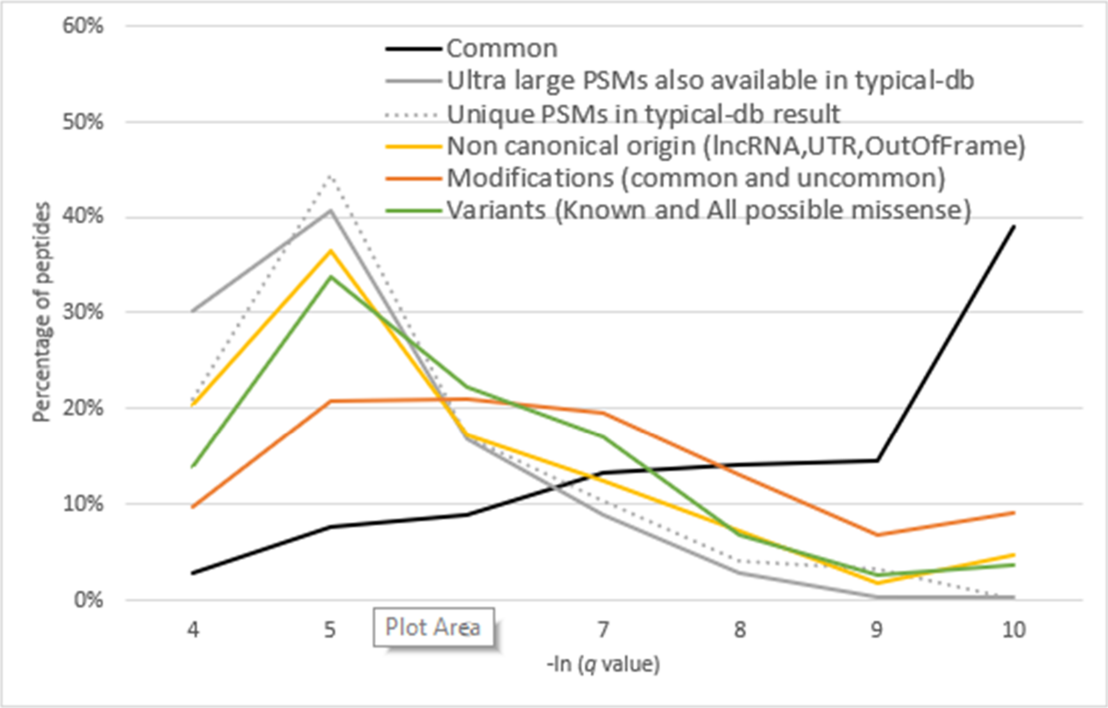

(c)

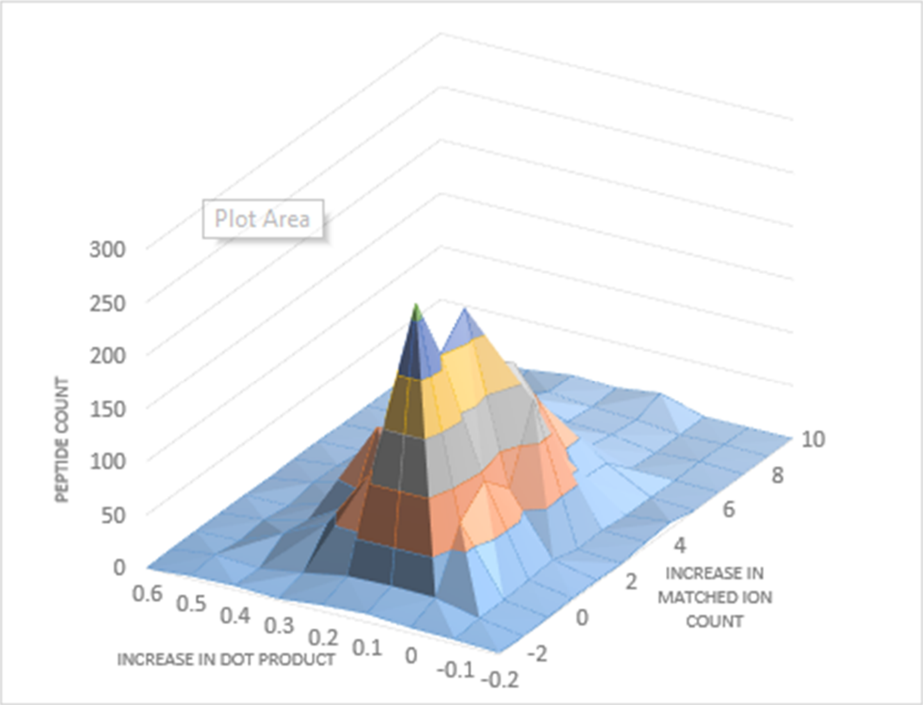

(d)

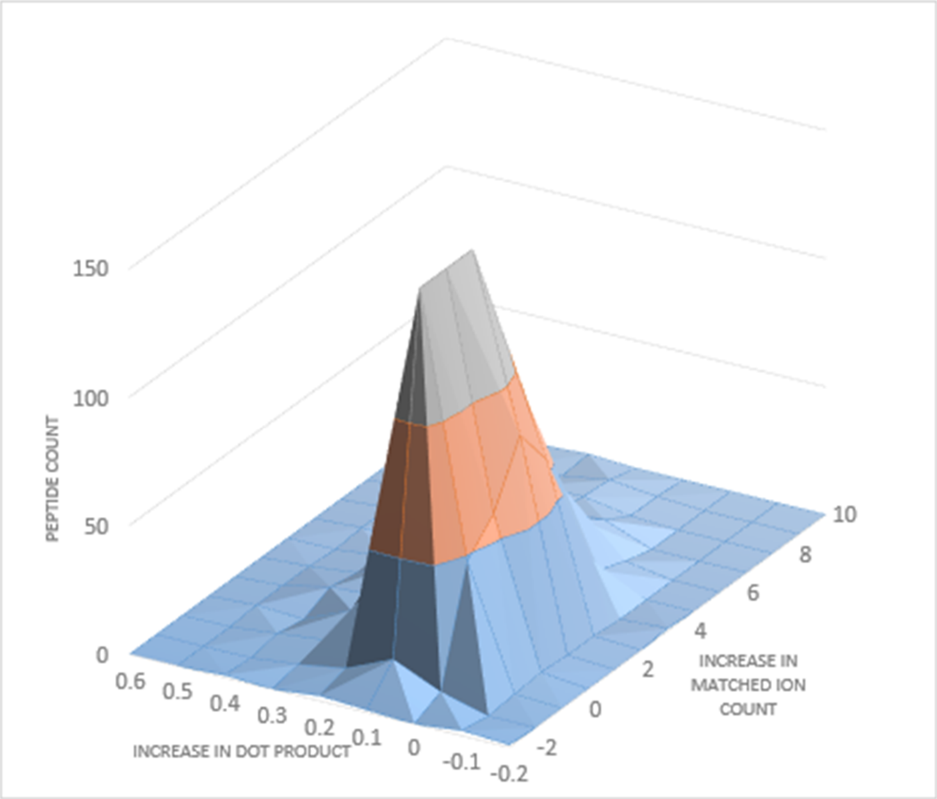

Figure S2. Comparison between canonical database search and ultra large database search. (**a**) Distribution of the q value for peptide IDs for Mel15 data set. 51,315 peptides that are common between Bolt's canonical database search and ultra large database results are shown in black, 5633 peptides that are unique to the ultra large database search but are also available to the canonical database search (shown in solid grey), 8950 peptides that are unique to the canonical database search (shown in dotted grey), 4702 peptides from non-canonical origins (lncRNA, Out of frame translation and UTRs, shown in yellow), 4984 peptides with modifications (both common and uncommon, shown in orange) and 1905 peptides with mutations (both known and all possible missense, shown in green). (**b**) Distribution of the *q* value for peptide IDs for OD5P data set. 23,900 peptides that are common between Bolt's canonical database search and ultra large database results

are shown in black, 5729 peptides that are unique to the ultra large database search but are also available to the canonical database search (shown in solid grey), 4548 peptides that are unique to the canonical database search (shown in dotted grey), 2395 peptides from non-canonical origins (lncRNA, Out of frame translation and UTRs, shown in yellow), 1947 peptides with modifications (both common and uncommon, shown in orange) and 781 peptides with mutations (both known and all possible missense, shown in green). (**c**) Increase in number of matched ion count and/or dot product observed for the Bolt's ultra-database search on Mel15 data (for the 2976 spectrum matches when canonical database search gave a different peptide ID). (**d**) Increase in number of matched ion count and/or dot product observed for the Bolt's ultra-database search on OD5P data (for the 1474 spectrum matches when canonical database search gave a different peptide ID).

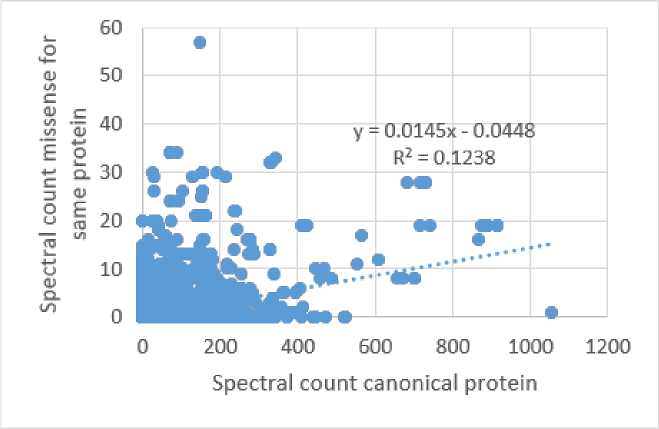

(**a**)

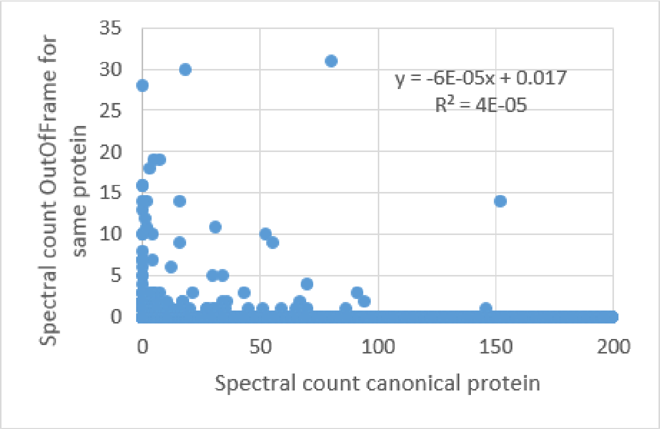

(**b**)

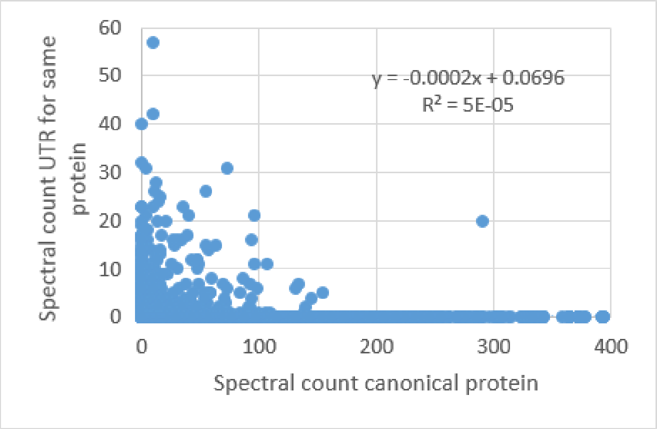

(**c**)

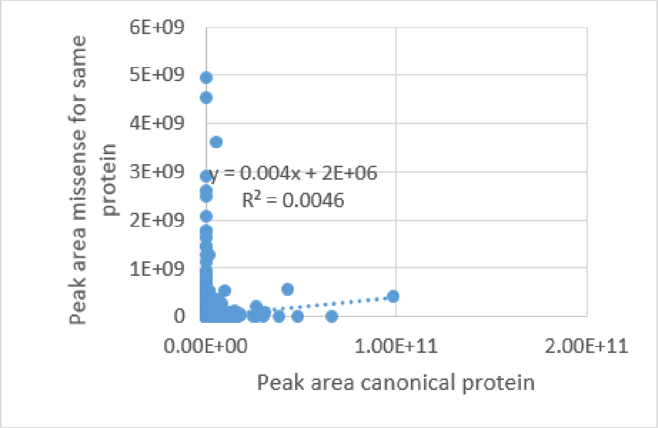

(**d**)

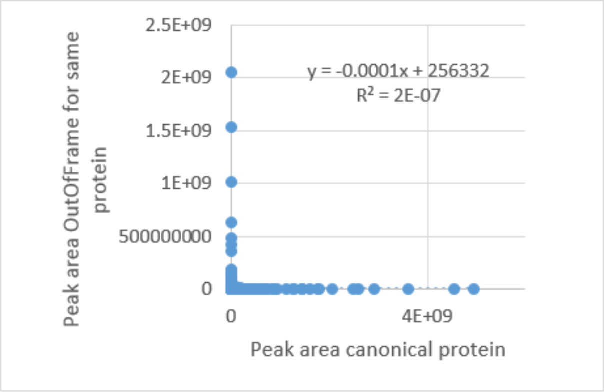

(**e**)

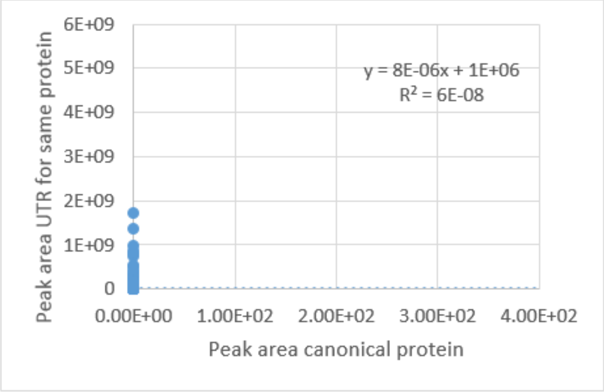

(**f**)

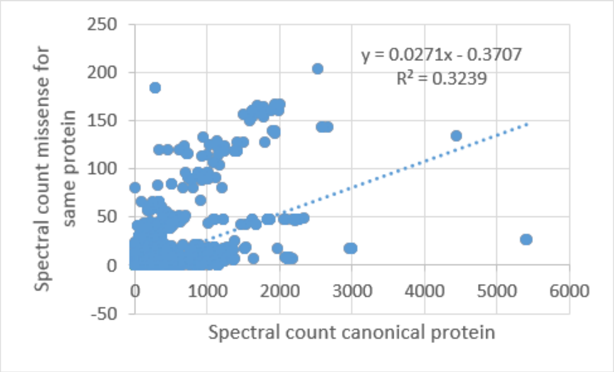

(**g**)

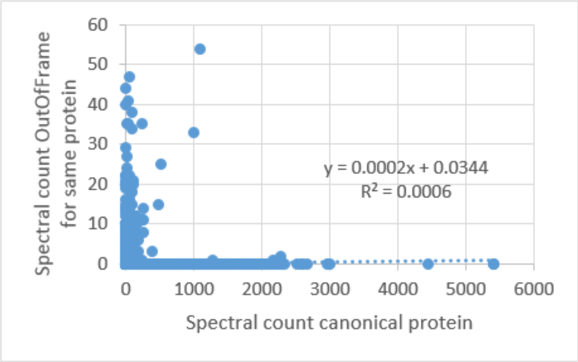

(**h**)

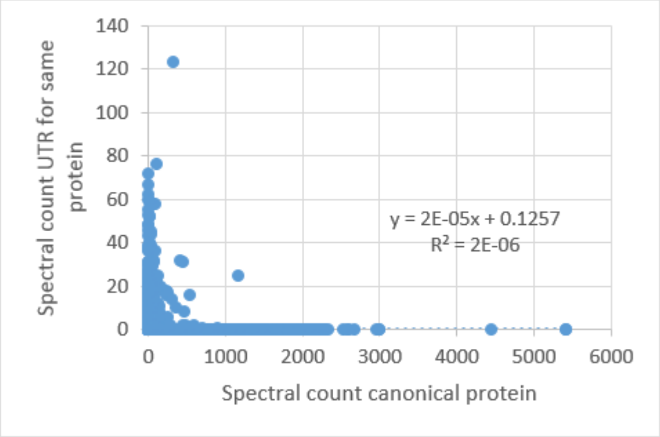

(**i**)

Figure S3. Abundance comparison between canonical coding region and non-canonical coding regions. (a) Abundance comparison between spectral count of canonical protein vs. spectral count of missense protein for same coding region for OD5P data set. (**b**) Abundance comparison between spectral count of canonical protein vs. spectral count of out of frame translation for same coding region for OD5P data set. (**c**) Abundance comparison between spectral count spectral count of canonical protein vs. spectral count of UTR for same coding region for OD5P data set. (**d**) Peak area of canonical protein vs. peak area of missense protein for same coding region for OD5P data set. (**e**) Peak area of canonical protein vs. peak area of out of frame translation for same coding region for OD5P data set. (**f**) Peak area of canonical protein vs. peak area of UTR for same coding region for OD5P data set. (**g**) Spectral count of canonical protein vs. spectral count of missense protein for same coding region for Mel15 data set. (**h**) Spectral count of canonical protein vs. spectral count of out of frame translation for same coding region for Mel15 data set. (**i**) Spectral count of canonical protein vs. spectral count of UTR for same coding region for Mel15 data set.

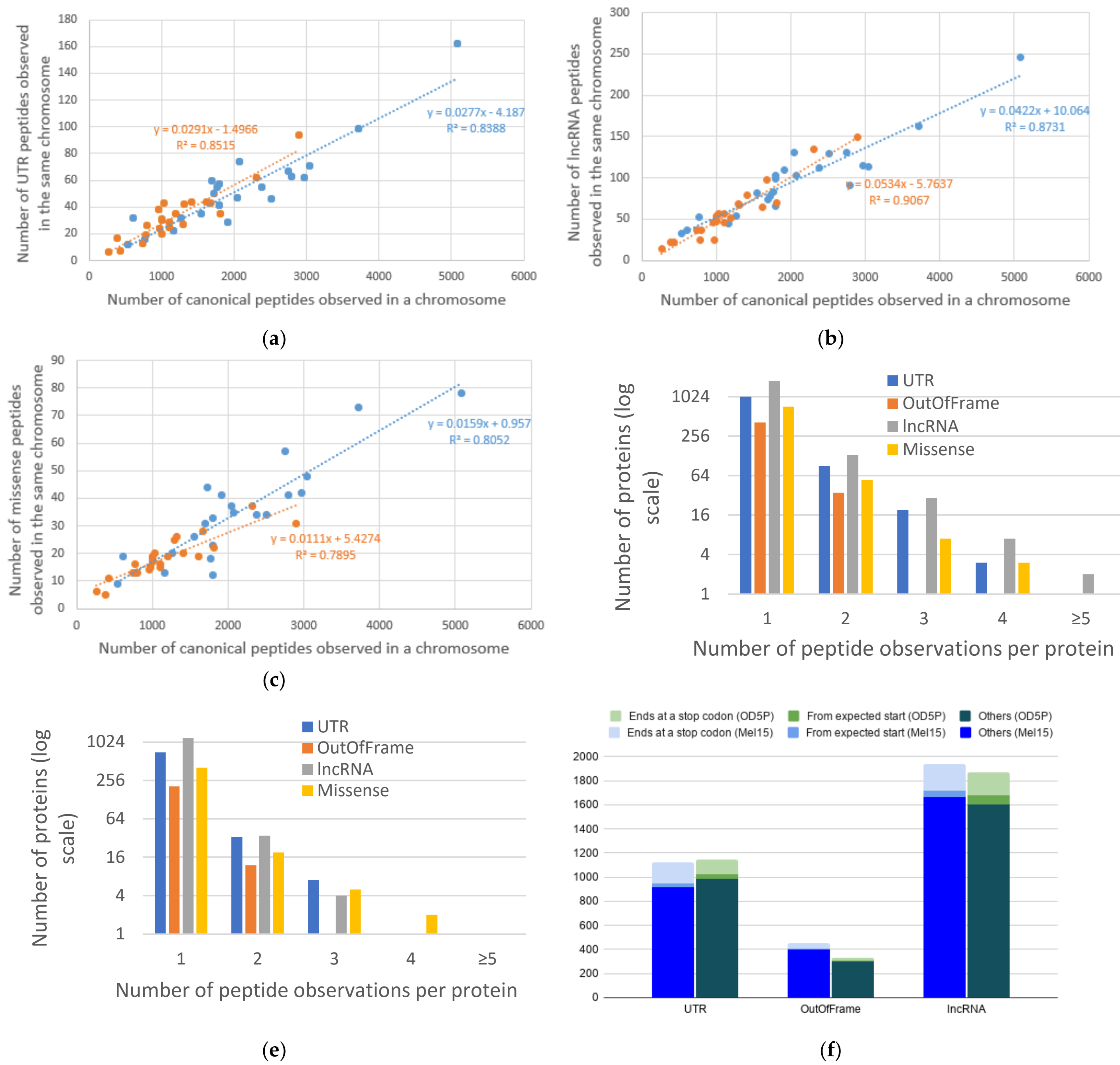

Figure S4. Study of chromosomal correlation for various non-canonical peptide IDs. Number of canonical peptides observed on a chromosome plotted against the number of (**a**) UTR (**b**) lncRNA (**c**) missense non-canonical peptides observed on the same chromosome for Mel15 (blue) and OD5P (orange) data sets. (**d**) Number of proteins plotted against the number of non-canonical peptides of various types observed for that protein for Mel15 data set. Y axis is logscale. (**e**) Number of proteins plotted against the number of non-canonical peptides of various types observed for that protein for Od5P data set. Y axis is logscale. (**f**) Number of non-canonical peptides observed for OD5P and Mel15 data set that start from the expected start codon, or end at a stop codon, or the remaining.

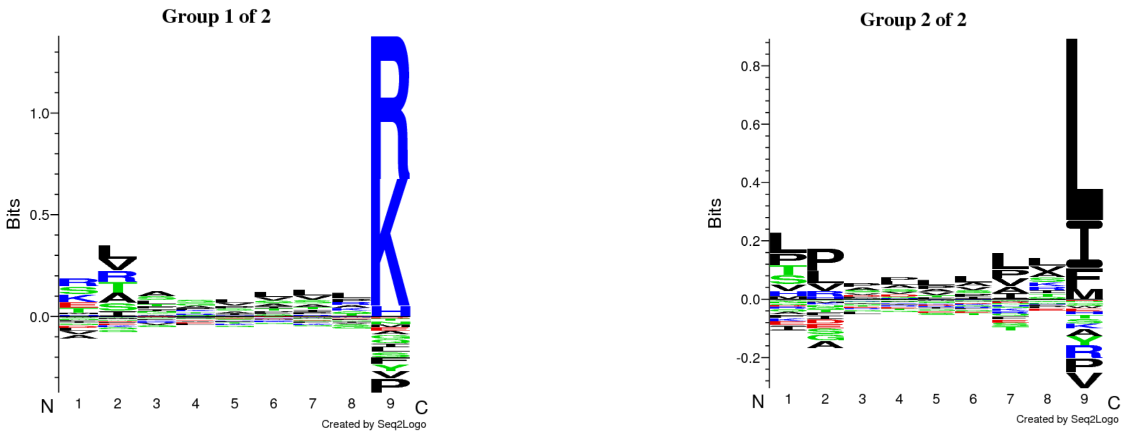

(**a**)

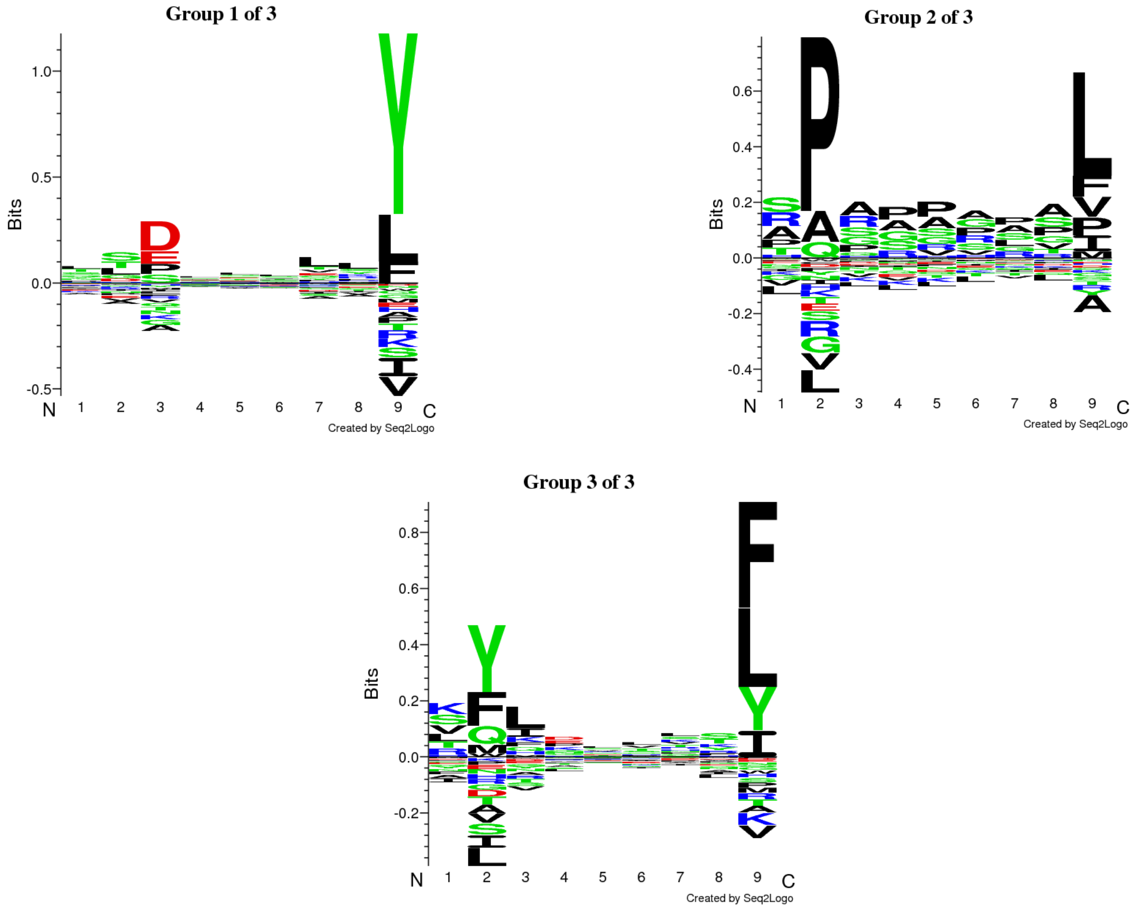

(**b**)

Figure S5. The Anchor motif predicted by Gibbs clustering approach for the non-canonical peptides of the two data sets. (**a**) For the Mel15 data set, Gibbs clustering produced 3 motif clusters of sizes 1703 and 1440. (**b**) For the OD5P data set, Gibbs clustering produced 4 clusters of sizes 663, 910 and 596.

(a)

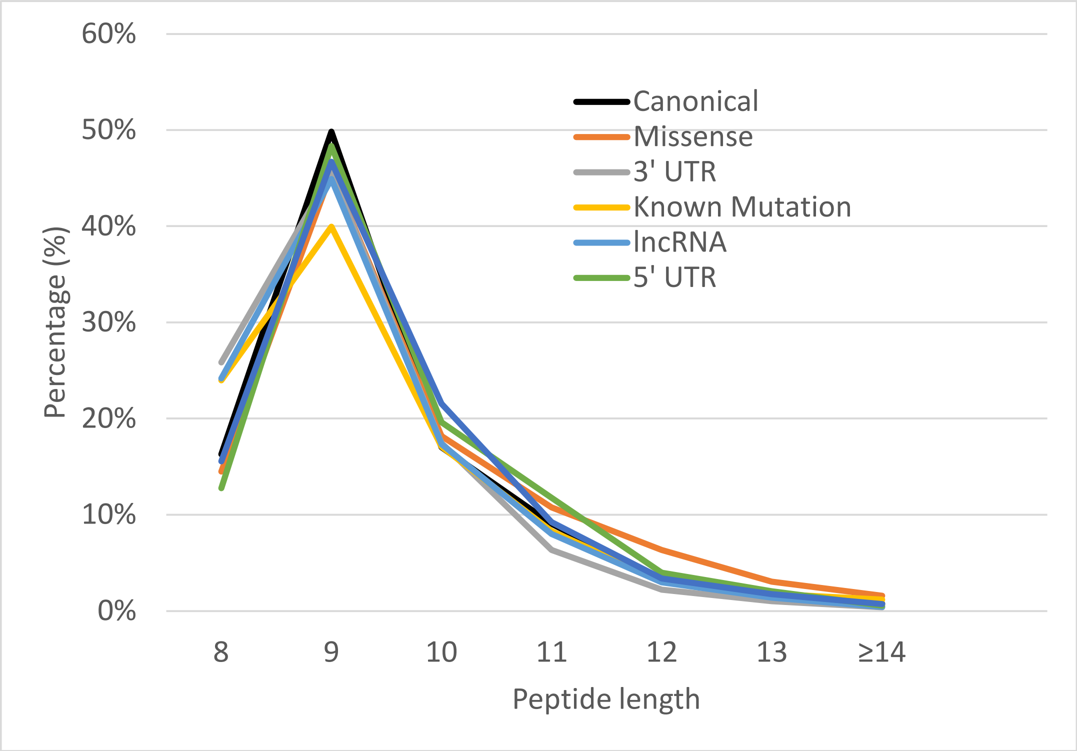

(b)

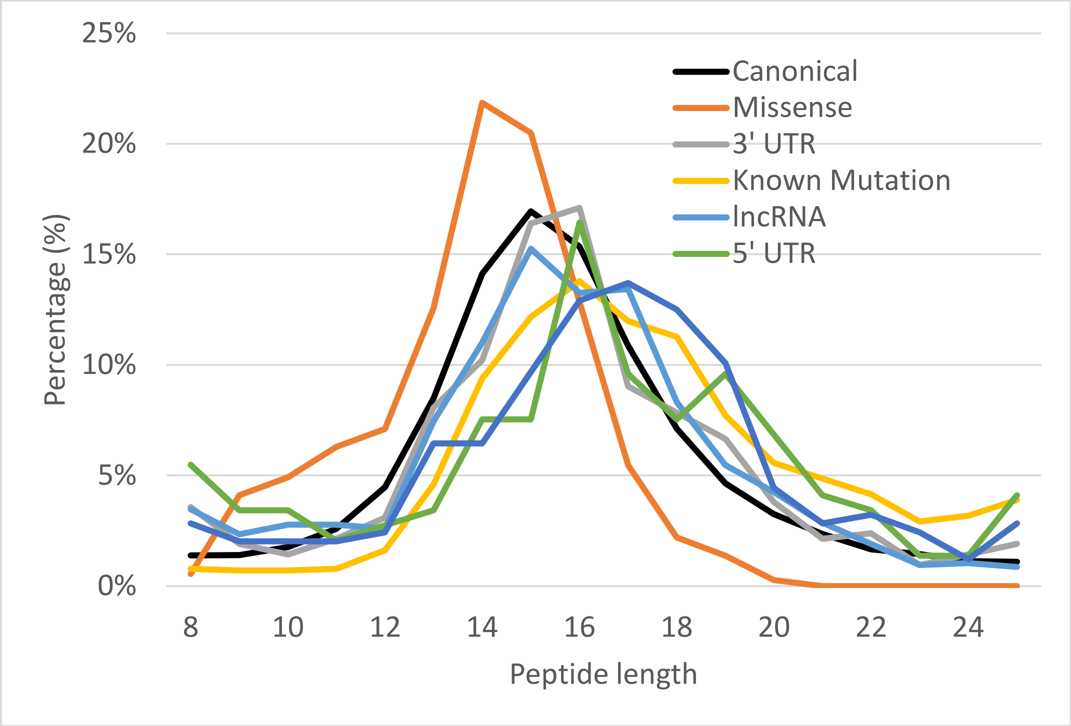

Figure S6. Peptide length distribution for various classes of peptides in (**a**) HLA-I and (**b**) HLA-II data sets.

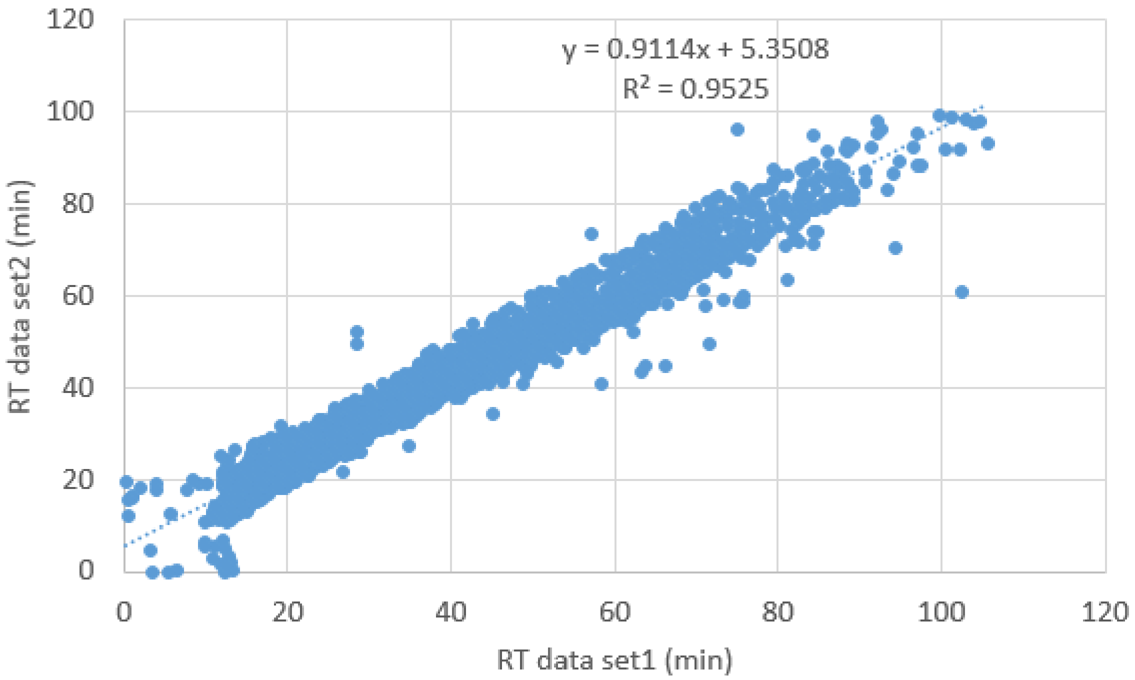

Figure S7. For non-canonical peptides that are observed in common across the two data sets (2016 and 2020), retention time observed in data set 1. is plotted against the retention time observed in data set 2.

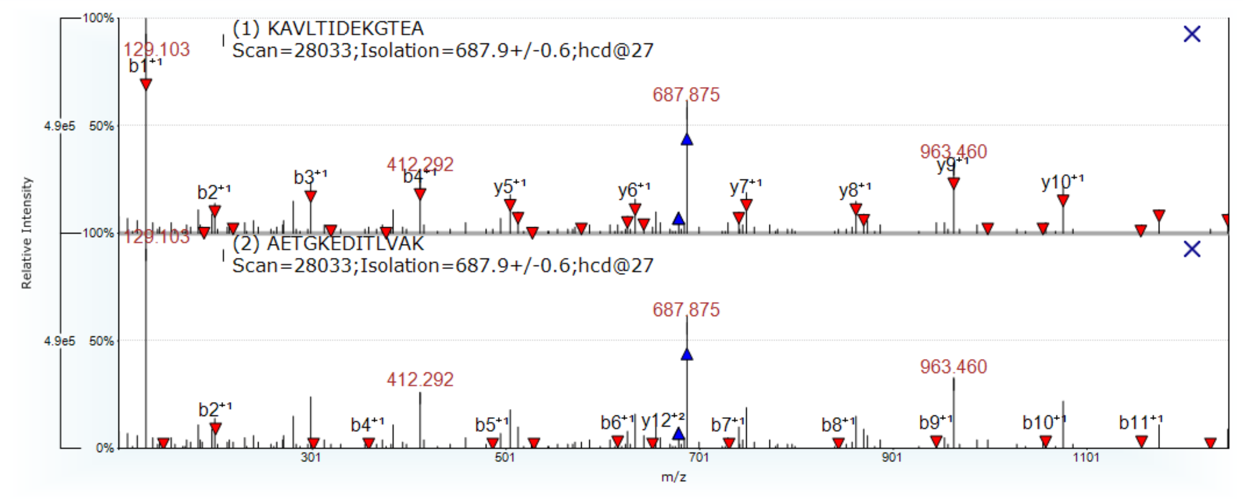

(a)

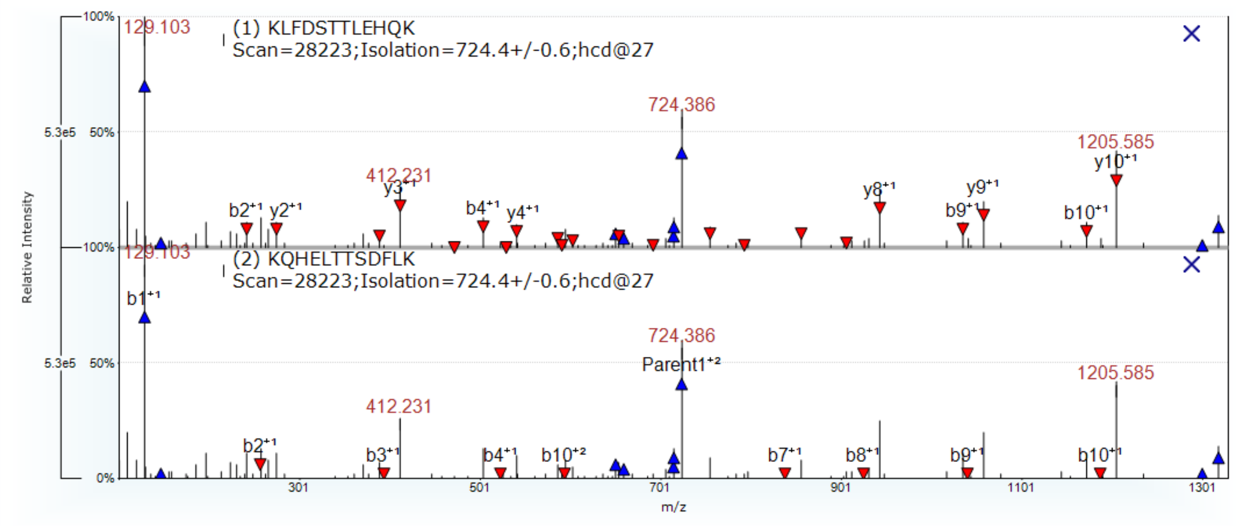

(b)

Figure S8. MS/MS spectra compared for forward and decoy peptide. Top panel of (**a**) shows peptide sequence KAVLTIDEKGTEA, from protein A1AT_HUMAN, having a strong match to scan number 28,033 in the raw file HLA-I-3A. The lower panel of (**a**) shows the same spectrum also having a rich fragment match to the reverse peptide AETGKEDITLVAK because many of the y-water ions from the target peptide are now the b ions of the reverse peptide. The top panel in (**b**) show peptide KLFDSTTLEHQK (from protein EIF3I_HUMAN) and (**b**) its reverse counterpart KQHELTTSDFLK, both having a good match to the scan number 28,223 from the same raw file.

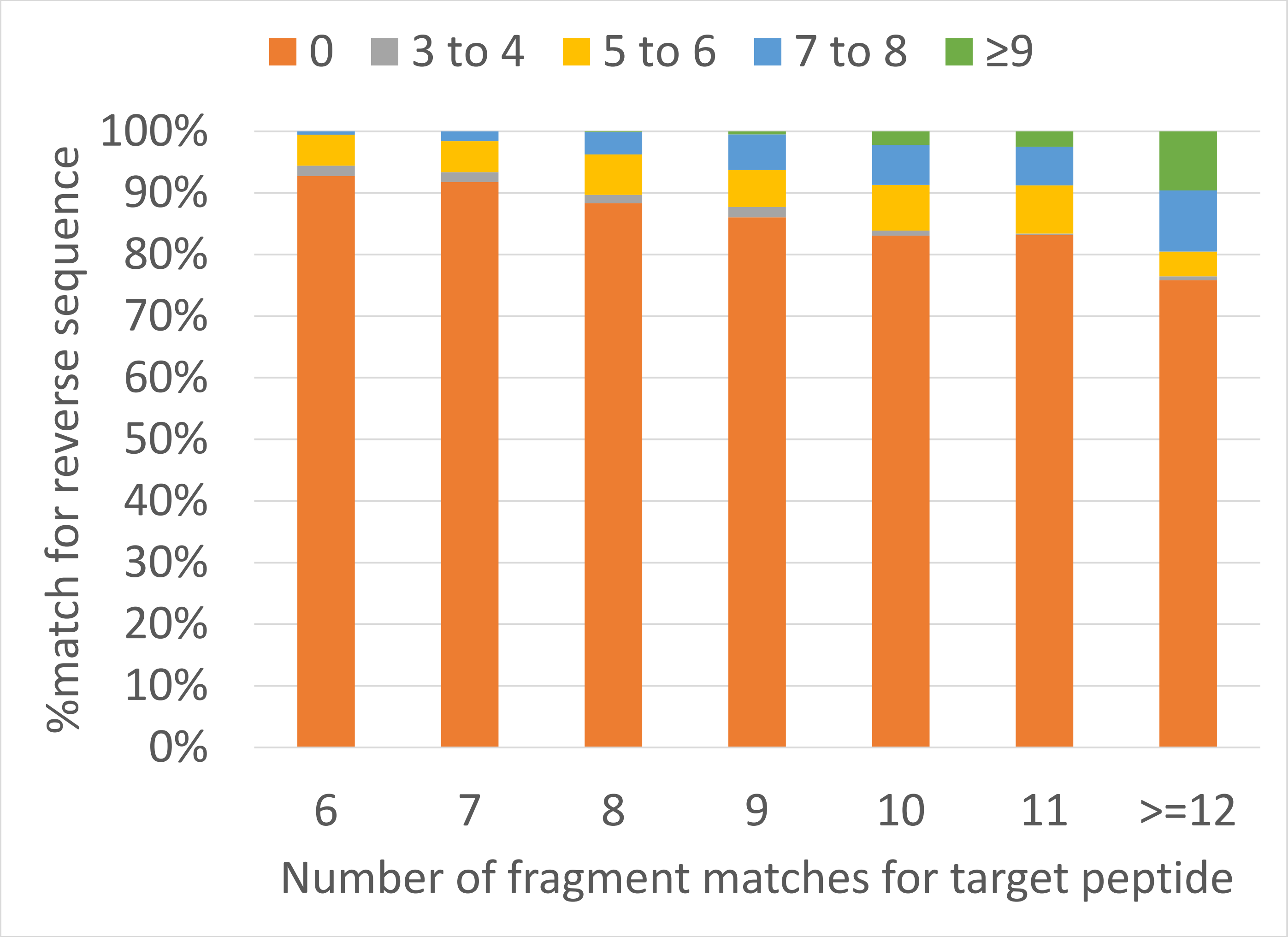

Figure S9. Distribution of number of fragments matched for the reverse peptide to the same spectrum for which the target peptide had a good match (for raw file HLA-I-3A). For target peptides having fewer fragments (e.g., 4 or 5) matched to a spectrum, the reverse peptide has almost no matches to the peptide (large orange or grey bar). But for target peptides that have a rich fragmentation match to a spectrum (e.g., ≥12), a significant number of those also have the reverse peptide with a rich fragmentation match to the same spectrum.

Table S1. Mutations reported by the original study using proteogenomics approach, and their finding using Bolt's approach.

Patient	Gene Name	Mutation	Peptide	MaxQuant FDR (Original Study)	Bolt's Result
Mel15	SYTL4	S363F	GRIAFFLKY	1%	Identified as missense at <1% FDR. GRIAFS(S->F)LKY
Mel15	RBPMS	P46L	RLFKGYEGSLIK	1%	Identified as missense at <0.1% FDR. RP(P->Xle)FKGYEGSLIK
Mel15	SEC23A	P52L	LPIQYEPVL	1%	Identified as missense at <0.1% FDR. P(P->Xle)PIQYEPVL
Mel15	H3F3C	T4I	RLKGATARK	5%	Identified as 5UTR expression at <0.1% FDR. Also a known cancer mutation RT(T->I)KQTARK
Mel15	NCAPG2	P333L	KLILWRGLK	1%	Identified as missense at <0.1% FDR. KP(P->Xle)ILWRGLK
Mel15	AKAP6	M1482I	KLKLPIIMK	1%	Identified as missense at <0.1% FDR. Matches two peptides: KLKLPM(M->Xle)IMK and KLKLPT(T->Xle)IMK
Mel15	MAP3K9	E689K	ASWVVPIDIK	5%	Not found. MS/MS matches better with out of frame translation for peptide KLWDPLDLK.
Mel15	ABCC2	S1342F	GRTGAGKSFL	5%	Not found. MS/MS matches with canonical peptide GRTGQKFSL (also isobaric).
Mel8	NOP16	P169L	SPGPVKLEL	5%	Identified as missense at <0.1% FDR.
Mel5	GABPA	E161K	ETSKQVTRW	5%	Identified as missense at <0.1% FDR.
Mel5	SEPT2	Q125R	YIDERFERY	5%	Identified as missense at <0.1% FDR.

Table S2. Theoretical fragments of the peptide PKAEFAEV from the protein sequence Serum Albumin, and the corresponding peptide from the reverse protein sequence. Both of these peptides have the exact same precursor mass (mol. wt. 889.455). y-water ions from the target peptide are the same as b- ions of the reverse peptide (marked as red). Also, b ions from the target peptide are exactly the same as y-water ions of the reverse peptide.

(a) Peptide from target sequence			
**b**	**Residues**	**y**	**y-water**
98.059	P	890.461	872.451
226.154	K	793.408	775.398
297.192	A	665.314	647.303
426.234	E	594.276	576.266
573.303	F	465.234	447.223
644.340	A	318.165	300.155
773.382	E	247.128	229.118
872.451	V	118.086	100.075
(b) Peptide from reverse sequence			
**b**	**Residues**	**y**	**y-water**
100.075	V	890.461	872.451
229.118	E	791.393	773.382
300.155	A	662.350	644.340
447.223	F	591.313	573.303
576.266	E	444.245	426.234
647.303	A	315.202	297.192
775.398	K	244.165	226.154
872.451	P	116.070	98.059

Additional References

1. Ma, B.; Zhang, K.; Hendrie, C.; Liang, C.; Li, M.; Doherty-Kirby, A.; Lajoie, G. PEAKS: powerful software for peptidede novo sequencing by tandem mass spectrometry. *Rapid Commun. Mass Spectrom.* **2003**, *17*, 2337–2342, doi:10.1002/rcm.1196.
2. The, M.; MacCoss, M.J.; Noble, W.S.; Käll, L. Fast and Accurate Protein False Discovery Rates on Large-Scale Proteomics Data Sets with Percolator 3.0. *J. Am. Soc. Mass Spectrom.* **2016**, *27*, 1719–1727, doi:10.1007/s13361-016-1460-7.
